# A Generalization of the Concavity of Rényi Entropy Power

**DOI:** 10.3390/e23121593

**Published:** 2021-11-27

**Authors:** Laigang Guo, Chun-Ming Yuan, Xiao-Shan Gao

**Affiliations:** 1 Laboratory of Mathematics and Complex Systems (Ministry of Education), School of Mathematical Sciences, Beijing Normal University, Beijing 100875, China; lgguo@bnu.edu.com; 2 KLMM, Academy of Mathematics and Systems Science, Chinese Academy of Sciences, Beijing 100190, China; cmyuan@mmrc.iss.ac.cn; 3 University of Chinese Academy of Sciences, Beijing 100049, China

**Keywords:** Rényi entropy, entropy power inequality, nonlinear heat equation

## Abstract

Recently, Savaré-Toscani proved that the Rényi entropy power of general probability densities solving the *p*-nonlinear heat equation in $R^{n}$ is a concave function of time under certain conditions of three parameters n,p,μ , which extends Costa’s concavity inequality for Shannon’s entropy power to the Rényi entropy power. In this paper, we give a condition Φ(n,p,μ) of n,p,μ under which the concavity of the Rényi entropy power is valid. The condition Φ(n,p,μ) contains Savaré-Toscani’s condition as a special case and much more cases. Precisely, the points (n,p,μ) satisfying Savaré-Toscani’s condition consist of a two-dimensional subset of $R^{3}$ , and the points satisfying the condition Φ(n,p,μ) consist a three-dimensional subset of $R^{3}$ . Furthermore, Φ(n,p,μ) gives the necessary and sufficient condition in a certain sense. Finally, the conditions are obtained with a systematic approach.

## 1. Introduction

In 1948, Claude Elwood Shannon [1] first introduced his mathematical theory of information. In particular, he presented the concept of entropy as a measure for information. On this foundation, Alfréd Rényi [2] then built one of his contributions in 1961. At the center, he introduced a new notion of entropy that included that of Shannon as a special case, and this is called Rényi entropy.

The *p-th Rényi entropy* [3,4] of a probability density function $f:R^{n}\to R$ is defined as

(1) $$\begin{array}{c} H_{p}\left(f\right):=\frac{1}{1-p}\log\int_{R^{n}}f^{p}\left(x\right)dx, \end{array}$$

for 0<p<+∞ , p≠1 . The *p*-th Rényi entropy power is given by

(2) $$\begin{array}{c} N_{p}\left(f\right):=\exp\left(\frac{\mu }{n}H_{p}\left(f\right)\right), \end{array}$$

where μ is a real-valued parameter. The Rényi entropy for p=1 is defined as the limit of $H_{p}\left(f\right)$ as p→1 . It follows from definition (1) that

$$\begin{array}{c} H_{1}\left(f\right)=\lim_{p\to 1}H_{p}\left(f\right)=-\int_{R^{n}}f\left(x\right)\log f\left(x\right)dx, \end{array}$$

which is Shannon’s entropy. Thus, the Rényi entropy power of index p=1,μ=2 , given by (2). coincides with Shannon’s entropy power

(3) $$\begin{array}{c} N_{1}\left(f\right):=\exp\left(\frac{2}{n}H_{1}\left(f\right)\right). \end{array}$$

Shannon’s *entropy power inequality (EPI)* is one of the most important information inequalities [1], which has many proofs, generalizations, and applications [5,6,7,8,9,10,11,12,13]. In particular, Costa presented a stronger version of the EPI in his seminal paper [14].

Let $X_{t}≜X+N_{n}\left(0,tI\right)$ be the *n*-dimensional random vector introduced by Costa [14,15,16,17] and $u(x_{t})$ the *probability density* of $X_{t}$ , which solves the heat equation in the whole space $R^{n}$ ,

(4) $$\begin{array}{c} \frac{\partial }{\partial t}u\left(x_{t}\right)=\Delta u\left(x_{t}\right). \end{array}$$

*Costa’s differential entropy* is defined to be

(5) $$H\left(u\left(x_{t}\right)\right)=-\int_{R^{n}}u\left(x_{t}\right)\log u\left(x_{t}\right)dx_{t}.$$

Related to EPI, Costa [14] proved that the *Shannon entropy power* $N\left(u\right)=\frac{1}{2\pi e}e^{\left(2/n)H(u\right)}$ is a concave function in *t*; that is, (d/dt)N(u)≥0 and $\left(d^{2}/d^{2}t\right)N\left(u\right)\leq 0$ . Several new proofs and generalizations for Costa’s EPI were given in [18,19,20,21].

Savaré-Toscani [22] proved that the *concavity of entropy power* is a property which is not restricted to the Shannon entropy power (3) in connection with the heat Equation (4), but it holds for the *p*-th Rényi entropy power (2). They put it in connection with the solution to the *nonlinear heat equation*

(6) $$\begin{array}{c} \frac{\partial }{\partial t}u\left(x_{t}\right)=\Delta u{\left(x_{t}\right)}^{p} \end{array}$$

posed in the whole space $R^{n}$ and $p\in R_{>0}$ and show that $\frac{d}{dt}N_{p}\left(u\right)\geq 0$ and $\frac{d^{2}}{d^{2}t}N_{p}\left(u\right)\leq 0$ hold if n,p,μ satisfy certain conditions.

In this paper, we give a generalization for the *concavity of the p-th Rényi entropy power (CREP)*. Precisely, we give a propositional logic formula Φ(n,p,μ) such that if n∈N,p,μ∈R satisfy this formula, then the CREP holds. The condition Φ(n,p,μ) extends the parameter range of the CREP given by Savaré-Toscani [22] and contains many more cases. Precisely, the points (n,p,μ) satisfying the condition given in [22] consist of a two-dimensional subset of $R^{3}$ and the points satisfying the condition Φ(n,p,μ) consist of a three-dimensional subset of $R^{3}$ . Furthermore, Φ(n,p,μ) gives the necessary and sufficient condition for CREP to be valid in a certain sense.

The formula Φ is obtained using a systematic procedure which can be considered as a parametric version of that given in [15,16,17,23], where parameters n,p,μ exist in the formulas. The procedure reduces the proof of the CREP to check the semi-positiveness of a quadratic form whose coefficients are polynomials in the parameters n,p,μ . In principle, a necessary and sufficient condition for the parameters to satisfy this property can be computed with the quantifier elimination [24]. In this paper, the problem is in a special form and an explicit proof is given.

The rest of this paper is organized as follows. In Section 2, we give the proof procedure and prove the concavity of entropy powers in the parametric case. In Section 3, we present the generalized version of CREP using the proof procedure. In Section 4, conclusions are presented.

## 2. Proof Procedure

In this section, we present a procedure to prove the CREP. To make the paper concise, we only give those steps that are needed in this paper.

### 2.1. Notations

Let $x_{t}=\left[x_{1,t},x_{2,t},\ldots ,x_{n,t}\right]$ be a set of variables depending on *t* and

$$\begin{array}{c} d^{\left(i\right)}x_{t}=dx_{1,t}dx_{2,t}\ldots dx_{i-1,t}dx_{i+1,t}\ldots dx_{n,t},i=1,2\ldots ,n. \end{array}$$

Let ${\left[n\right]}_{0}=\left\{0,1,\ldots ,n\right\}$ and [n]={1,…,n} . To simplify the notations, we use *u* to denote $u(x_{t})$ in the rest of the paper. Denote

$$P_{n}=\cup_{h=0}^{\infty }P_{h,n},\;P_{h,n}=\left\{\frac{\partial^{h}u}{\partial^{h_{1}}x_{1,t}\cdots \partial^{h_{n}}x_{n,t}}:h=\sum_{i=1}^{n}h_{i},h_{i}\in N\right\}$$

as the set of all derivatives of *u* with respect to the differential operators $\frac{\partial }{\partial x_{i,t}},i=1,\ldots ,n$ , R[n,p,μ] as the set of polynomials in parameters n,p,μ , and

$$R=R\left[n,p,\mu \right]\left[P_{n}\right]$$

as the set of polynomials in $P_{n}$ with coefficients in R[n,p,μ] . For $v\in P_{h,n}$ , we say *v* has order ord(v)=h . For a monomial $\prod_{i=1}^{r}v_{i}^{d_{i}}$ with $v_{i}\in P_{n}$ , its *degree*, *order*, and *total order* are defined to be $\sum_{i=1}^{r}d_{i}$ , $\max_{i=1}^{r}\mathrm{ord}\left(v_{i}\right)$ , and $\sum_{i=1}^{r}d_{i}\cdot \mathrm{ord}\left(v_{i}\right)$ , respectively.

A polynomial in R is called a *k*th-order *differentially homogenous polynomial* or simply a *k*th-order *differential form*, if all its monomials have degree *k* and total order *k*. Let $M_{k,n}$ be the set of all monomials which have degree *k* and total order *k*. Then, the set of *k*th-order differential forms is an R -linear vector space generated by $M_{k,n}$ , which is denoted as ${\mathrm{Span}}_{R}\left(M_{k,n}\right)$ . We use Gaussian elimination in ${\mathrm{Span}}_{R}\left(M_{k,n}\right)$ by treating the monomials as variables. We always use the *lexicographic order for the monomials* defined in [15,16,17].

### 2.2. Sketch of the Proof

In this section, we give the procedure to prove the *CREP*. The property $\frac{d}{dt}N_{p}\left(u\right)\geq 0$ can be easily proved [22]. We focus on proving $\frac{d^{2}}{d^{2}t}N_{p}\left(u\right)\leq 0$ . The procedure consists of four steps.

In step 1, we reduce the proof of *CREP* into the proof of an integral inequality, as shown by the following lemma, the proof of which is given in Section 2.4.

**Lemma** **1.** *The proof of $\frac{d^{2}}{d^{2}t}N_{p}\left(u\right)\leq 0$ can be reduced to show*

(7) $$\begin{array}{c} \int_{R^{n}}u^{3p-6}E_{2,n}dx_{t}\geq 0, \end{array}$$

*under the condition $p\geq 1-\frac{\mu }{n}$ , where $E_{2,n}=\sum_{a=1}^{n}\sum_{b=1}^{n}E_{2,n,a,b}$ is a fourth-order differential form in $R\left[n,p,\mu \right]\left[V_{a,b}\right]$ and*

(8) $$V_{a,b}=\left\{\frac{\partial^{h}u}{\partial^{h_{1}}x_{a,t}\partial^{h_{2}}x_{b,t}}:h\in {\left[3\right]}_{0};a,b\in \left[n\right]\right\}.$$

In step 2, we compute the constraints, which are relations satisfied by the probability density *u* of $X_{t}$ . Since $E_{2,n}$ in (7) is a fourth-order differential form, we need only the constraints which are fourth-order differential forms. A fourth-order differential form *R* is called an *equational or inequality constraint* if

(9) $$\int_{R^{n}}u^{3p-6}Rdx_{t}=0\;\mathrm{or}\;\int_{R^{n}}u^{3p-6}Rdx_{t}\geq 0.$$

The method to compute the constraints is given in Section 2.3. Suppose that the equational and inequality constraints are respectively

(10) $$\begin{array}{c} C_{E}=\left\{R_{i},\;|\;i=1,\ldots ,N_{1}\right\}, \end{array}$$

(11) $$\begin{array}{c} C_{I}=\left\{I_{i},\;|\;i=1,\ldots ,N_{2}\right\}. \end{array}$$

In step 3, we find a propositional formula Φ(n,p,μ) such that when n∈N and p,μ∈R satisfy Φ ,

(12) $$\begin{array}{c} \exists c_{j},e_{i}\in R,s.t.\;\;E_{2,n}-\sum_{i=1}^{N_{1}}e_{i}R_{i}-\sum_{j=1}^{N_{2}}c_{j}I_{j}=S\geq 0\;\mathrm{and}\;c_{j}\geq 0,j=1,\ldots ,N_{2} \end{array}$$

where *S* is a sum of squares (SOS). Details of this step and the formula Φ(n,p,μ) are given in Section 3.

To summarize the proof procedure, we have

**Theorem** **1.** *The CREP is true if Φ(n,p,μ) is valid.*

**Proof.**  By Lemma 1, we have the following proof for CREP:

(13) $$\begin{array}{cc} \; & \int_{R}u^{3p-6}E_{2,n}dx_{t}\\ \overset{\left(12\right)}{=} & \int_{R}u^{3p-6}\left(\sum_{i=1}^{N_{1}}e_{i}R_{i}+\sum_{j=1}^{N_{2}}c_{j}I_{j}+S\right)dx_{t}\\ \overset{S1}{=} & \int_{R}u^{3p-6}\left(\sum_{j=1}^{N_{2}}c_{j}I_{j}+S\right)dx_{t}\\ \overset{S2}{\geq } & \int_{R}u^{3p-6}Sdx_{t}\overset{S3}{\geq }0. \end{array}$$

Equality S1 is true, because $R_{i}$ are equational constraints. Inequality S2 is true, because $I_{j}$ are inequality constraints. Inequality S3 is true, because *S* is an SOS and hence S≥0 under the condition Φ(n,p,μ) . □

### 2.3. The Equational Constraints

In this section, we show how to find the second-order equational constraints. A *second-order equational constraint* is a fourth-order differential form in $R\left[n,p,\mu \right]\left[P_{2,n}\right]$ such that $\int_{R^{n}}\;u^{3p-6}R\;dx_{t}=0$ . We need the following property.

**Property** **1.** *Let $a,r,m_{i},k_{i}\in N_{>0}$ and $u^{\left(m_{i}\right)}$ be an $m_{i}$ th-order derivative of u. If $u(x_{t})$ is a smooth, strictly positive and rapidly decaying probability density, then*

(14) $$\begin{array}{c} \int_{-\infty }^{\infty }\ldots \int_{-\infty }^{\infty }u^{3p-2}\left[\prod_{i=1}^{r}\frac{{\left[u^{\left(m_{i}\right)}\right]}^{k_{i}}}{u^{k_{i}}}\right]{|}_{x_{a,t}=-\infty }^{\infty }d^{\left(a\right)}x_{t}=0, \end{array}$$

*with* $\sum_{i=1}^{r}k_{i}m_{i}=4,\;\sum_{i=1}^{r}k_{i}=4$ .

When p≥2 , Property 1 follows from [25]. While 0<p<2,p≠1 , we make the assumption that $u(x_{t})$ also satisfies Property 1.

Using Property 1, we can compute 28 second-order equational constraints using the method given in [15,16,17]:

(15) $$C_{2,n}=\left\{R_{i,a,b}\;:\;i=1,\ldots ,28\right\}\subset R\left[n,p,\mu \right]\left[V_{a,b}\right],$$

where $R_{i,a,b}$ can be found in the Appendix A. Note that a,b are variables taking values in [n] .

We use an example to show how to obtain these constraints. Starting from a monomial $u\frac{\partial^{2}u}{\partial^{2}x_{a,t}}{\left(\frac{\partial u}{\partial x_{a,t}}\right)}^{2}$ with degree 4 and total order 4, using integral by parts, we have

(16) $$\begin{array}{c} \int u^{3p-6}u\frac{\partial^{2}u}{\partial^{2}x_{a,t}}{\left(\frac{\partial u}{\partial x_{a,t}}\right)}^{2}dx_{t}\\ =\int_{-\infty }^{\infty }\ldots \int_{-\infty }^{\infty }\left[u^{3p-5}\frac{\partial u}{\partial x_{a,t}}{\left(\frac{\partial u}{\partial x_{a,t}}\right)}^{2}\right]{|}_{x_{a,t}=-\infty }^{\infty }d^{\left(a\right)}x_{t}\\ -\int \frac{\partial u}{\partial x_{a,t}}\left[\frac{\partial }{\partial x_{a,t}}\left(u^{3p-5}{\left(\frac{\partial u}{\partial x_{a,t}}\right)}^{2}\right)\right]dx_{t}\\ \overset{\left(14\right)}{=}-\int \frac{\partial u}{\partial x_{a,t}}\left[\frac{\partial }{\partial x_{a,t}}\left(u^{3p-5}{\left(\frac{\partial u}{\partial x_{a,t}}\right)}^{2}\right)\right]dx_{t}. \end{array}$$

Then,

(17) $$\begin{array}{c} \int u^{3p-6}u\frac{\partial^{2}u}{\partial^{2}x_{a,t}}{\left(\frac{\partial u}{\partial x_{a,t}}\right)}^{2}+\frac{\partial u}{\partial x_{a,t}}\left[\frac{\partial }{\partial x_{a,t}}\left(u^{3p-5}{\left(\frac{\partial u}{\partial x_{a,t}}\right)}^{2}\right)\right]dx_{t}\\ =\int u^{3p-6}\left[3p{\left(\frac{\partial u}{\partial x_{a,t}}\right)}^{4}+3u\frac{\partial^{2}u}{\partial^{2}x_{a,t}}{\left(\frac{\partial u}{\partial x_{a,t}}\right)}^{2}-5{\left(\frac{\partial u}{\partial x_{a,t}}\right)}^{4}\right]dx_{t}=0. \end{array}$$

We then obtain a 2th-order constraint: $R_{1,a,b}=3p{\left(\frac{\partial u}{\partial x_{a,t}}\right)}^{4}+3u\frac{\partial^{2}u}{\partial^{2}x_{a,t}}{\left(\frac{\partial u}{\partial x_{a,t}}\right)}^{2}-5{\left(\frac{\partial u}{\partial x_{a,t}}\right)}^{4}$ . The other 27 constraints in $C_{2,n}$ are obtained in the same way.

### 2.4. Proof of Lemma 1

We first prove several lemmas.

**Lemma** **2.**
(18) $$\begin{array}{c} \frac{dH_{p}\left(u\right)}{dt}=\frac{p}{1-p}\frac{\int u^{p-1}\Delta u^{p}dx_{t}}{\int u^{p}dx_{t}}, \end{array}$$

(19) $$\frac{d^{2}H_{p}\left(u\right)}{d^{2}t}=\frac{p}{1-p}\frac{\frac{\partial }{\partial t}\left(\int u^{p-1}\frac{\partial u}{\partial t}dx_{t}\right)\int u^{p}dx_{t}-p{\left(\int u^{p-1}\frac{\partial u}{\partial t}dx_{t}\right)}^{2}}{{\left(\int u^{p}dx_{t}\right)}^{2}}.$$

**Proof.**  By the definition of *p*-Rényi entropy (1), we have

$$\begin{array}{cc} \frac{dH_{p}\left(u\right)}{dt} & =\frac{p}{1-p}\frac{\int u^{p-1}\frac{\partial u}{\partial t}dx_{t}}{\int u^{p}dx_{t}}=\frac{p}{1-p}\frac{\int u^{p-1}\Delta u^{p}dx}{\int u^{p}dx},\\ \frac{d^{2}H_{p}\left(u\right)}{d^{2}t} & =\frac{p}{1-p}\frac{\frac{\partial }{\partial t}\left(\int u^{p-1}\frac{\partial u}{\partial t}dx_{t}\right)\int u^{p}dx_{t}-\int u^{p-1}\frac{\partial u}{\partial t}dx_{t}\frac{\partial }{\partial t}\left(\int u^{p}dx_{t}\right)}{{\left(\int u^{p}dx_{t}\right)}^{2}}\\ \; & \;\;\;\;=\frac{p}{1-p}\frac{\frac{\partial }{\partial t}\left(\int u^{p-1}\frac{\partial u}{\partial t}dx_{t}\right)\int u^{p}dx_{t}-\int u^{p-1}\frac{\partial u}{\partial t}dx_{t}\int pu^{p-1}\frac{\partial u}{\partial t}dx_{t}}{{\left(\int u^{p}dx_{t}\right)}^{2}}\\ \; & \;\;\;\;=\frac{p}{1-p}\frac{\frac{\partial }{\partial t}\left(\int u^{p-1}\frac{\partial u}{\partial t}dx_{t}\right)\int u^{p}dx_{t}-p{\left(\int u^{p-1}\frac{\partial u}{\partial t}dx_{t}\right)}^{2}}{{\left(\int u^{p}dx_{t}\right)}^{2}}. \end{array}$$

□

**Lemma** **3.** *We have*

(20) $$\begin{array}{c} \int u^{p-1}\Delta u^{p}dx_{t}=\int \Delta u^{p-1}u^{p}dx_{t}. \end{array}$$

**Proof.**  Integrating by parts [22], we have

$$\int u^{p-1}\Delta u^{p}dx_{t}=-\int \nabla u^{p-1}\nabla u^{p}dx_{t}=\int \Delta u^{p-1}u^{p}dx_{t}.$$

□

By Cauchy–Schwarz inequality, we have

**Lemma 4.** 
(21) $$\begin{array}{c} {\left(\int \Delta u^{p-1}u^{p}dx_{t}\right)}^{2}\leq \int u^{p}dx_{t}\int {\left(\Delta u^{p-1}\right)}^{2}u^{p}dx_{t}. \end{array}$$

Then, we obtain

(22) $$\begin{array}{cc} \frac{d^{2}}{d^{2}t}N_{p}\left(u\right) & \;\;\;=\frac{\mu }{n}\frac{d^{2}H_{p}\left(u\right)}{d^{2}t}e^{\frac{\mu }{n}H_{p}\left(u\right)}+{\left(\frac{\mu }{n}\frac{dH_{p}\left(u\right)}{dt}\right)}^{2}e^{\frac{\mu }{n}H_{p}\left(u\right)}\\ \; & \;\;\;=\frac{\mu }{n}e^{\frac{\mu }{n}H_{p}\left(u\right)}I_{2,n}, \end{array}$$

where $I_{2,n}=\frac{d^{2}H_{p}\left(u\right)}{d^{2}t}+\frac{\mu }{n}{\left(\frac{dH_{p}\left(u\right)}{dt}\right)}^{2}$ . So, by (18), (19), we have

(23) $$\begin{array}{cc} I_{2,n} & =\frac{p}{1-p}\frac{\frac{\partial }{\partial t}\left(\int u^{p-1}\frac{\partial u}{\partial t}dx_{t}\right)\int u^{p}dx_{t}-p{\left(\int u^{p-1}\frac{\partial u}{\partial t}dx_{t}\right)}^{2}}{{\left(\int u^{p}dx_{t}\right)}^{2}}\\ \; & +\frac{\mu }{n}{\left(\frac{p}{1-p}\frac{\int u^{p-1}\Delta u^{p}dx_{t}}{\int u^{p}dx_{t}}\right)}^{2}\\ \; & =\frac{\mu p^{2}}{n{\left(1-p\right)}^{2}}\frac{{\left(\int u^{p-1}\Delta u^{p}dx_{t}\right)}^{2}}{{\left(\int u^{p}dx_{t}\right)}^{2}}+\frac{p}{1-p}\frac{\frac{\partial }{\partial t}\left(\int u^{p-1}\frac{\partial u}{\partial t}dx_{t}\right)\int u^{p}dx_{t}}{{\left(\int u^{p}dx_{t}\right)}^{2}}\\ \; & -\frac{p^{2}}{1-p}\frac{{\left(\int u^{p-1}\Delta u^{p}dx_{t}\right)}^{2}}{{\left(\int u^{p}dx_{t}\right)}^{2}}\\ \; & =\left(\frac{\mu p^{2}}{n{\left(1-p\right)}^{2}}-\frac{p^{2}}{1-p}\right)\frac{{\left(\int u^{p-1}\Delta u^{p}dx_{t}\right)}^{2}}{{\left(\int u^{p}dx_{t}\right)}^{2}}\\ \; & +\frac{p}{1-p}\frac{\frac{\partial }{\partial t}\left(\int u^{p-1}\frac{\partial u}{\partial t}dx_{t}\right)\int u^{p}dx_{t}}{{\left(\int u^{p}dx_{t}\right)}^{2}}\\ \; & \overset{\left(20\right)}{=}\frac{\left(\mu -n\left(1-p\right)\right)p^{2}}{n{\left(1-p\right)}^{2}}\frac{{\left(\int \Delta u^{p-1}u^{p}dx_{t}\right)}^{2}}{{\left(\int u^{p}dx_{t}\right)}^{2}}+\frac{p}{1-p}\frac{\frac{\partial }{\partial t}\left(\int u^{p-1}\frac{\partial u}{\partial t}dx_{t}\right)\int u^{p}dx_{t}}{{\left(\int u^{p}dx_{t}\right)}^{2}}\\ \; & \overset{\left(i\right)}{\leq }\frac{\left(\mu -n\left(1-p\right)\right)p^{2}}{n{\left(1-p\right)}^{2}}\frac{\int u^{p}dx_{t}\int {\left(\Delta u^{p-1}\right)}^{2}u^{p}dx_{t}}{{\left(\int u^{p}dx_{t}\right)}^{2}}\\ \; & +\frac{p}{1-p}\frac{\frac{\partial }{\partial t}\left(\int u^{p-1}\frac{\partial u}{\partial t}dx_{t}\right)\int u^{p}dx_{t}}{{\left(\int u^{p}dx_{t}\right)}^{2}}\\ \; & =\frac{1}{\int u^{p}dx_{t}}\left(\frac{\left(\mu -n\left(1-p\right)\right)p^{2}}{n{\left(1-p\right)}^{2}}\int {\left(\Delta u^{p-1}\right)}^{2}u^{p}dx_{t}+\frac{p}{1-p}\frac{\partial }{\partial t}\left(\int u^{p-1}\frac{\partial u}{\partial t}dx_{t}\right)\right)\\ \; & =\frac{1}{\int u^{p}dx_{t}}\int \left(\frac{\left(\mu -n\left(1-p\right)\right)p^{2}}{n{\left(1-p\right)}^{2}}{\left(\Delta u^{p-1}\right)}^{2}u^{p}+\frac{p}{1-p}\frac{\partial }{\partial t}\left(u^{p-1}\frac{\partial u}{\partial t}\right)\right)dx_{t}\\ \; & =\frac{1}{\int u^{p}dx_{t}}\int F_{2,n}dx_{t}, \end{array}$$

where $F_{2,n}=\frac{\left(\mu -n\left(1-p\right)\right)p^{2}}{n{\left(1-p\right)}^{2}}{\left(\Delta u^{p-1}\right)}^{2}u^{p}+\frac{p}{1-p}\frac{\partial }{\partial t}\left(u^{p-1}\frac{\partial u}{\partial t}\right)$ .

**Remark** **1.** *In (23), the step (i) is according to (21), and $\frac{\left(\mu -n\left(1-p\right)\right)p^{2}}{n{\left(1-p\right)}^{2}}\geq 0$ should be satisfied, which is true under condition $p\geq 1-\frac{\mu }{n}$ . When μ:=2+n(p−1) , $\frac{\left(\mu -n\left(1-p\right)\right)p^{2}}{n{\left(1-p\right)}^{2}}\geq 0$ yields $p\geq 1-\frac{1}{n}$ . Savaré-Toscani [22] also used the inequality (21), but ignore the nonnegativity of the coefficient $\frac{\left(\mu -n\left(1-p\right)\right)p^{2}}{n{\left(1-p\right)}^{2}}$ ; thus, the parameter’s range $p>1-\frac{2}{n}$ in [22] should be corrected to $p\geq 1-\frac{1}{n}$ .*

Furthermore, we have

(24) $$\begin{array}{cc} F_{2,n} & =\frac{\left(\mu -n\left(1-p\right)\right)p^{2}}{n{\left(1-p\right)}^{2}}u^{p}\sum_{a=1}^{n}\sum_{b=1}^{n}\left[\left(\frac{\partial^{2}}{\partial^{2}x_{a,t}}u^{p-1}\right)\left(\frac{\partial^{2}}{\partial^{2}x_{b,t}}u^{p-1}\right)\right]\\ & +\frac{p}{1-p}\frac{\partial }{\partial t}\left[u^{p-1}\sum_{a=1}^{n}\left(\frac{\partial^{2}}{\partial^{2}x_{a,t}}u^{p}\right)\right]\\ \; & =\frac{\left(\mu -n\left(1-p\right)\right)p^{2}}{n{\left(1-p\right)}^{2}}u^{p}\sum_{a=1}^{n}\sum_{b=1}^{n}\left[\left(\frac{\partial^{2}}{\partial^{2}x_{a,t}}u^{p-1}\right)\left(\frac{\partial^{2}}{\partial^{2}x_{b,t}}u^{p-1}\right)\right]\\ \; & +\frac{p}{1-p}\sum_{a=1}^{n}\left[\left(p-1\right)u^{p-2}\left(\frac{\partial^{2}}{\partial^{2}x_{a,t}}u^{p}\right)\frac{\partial u}{\partial t}+pu^{p-1}\frac{\partial^{2}}{\partial^{2}x_{a,t}}\left(u^{p-1}\frac{\partial u}{\partial t}\right)\right]\\ \; & =\frac{\left(\mu -n\left(1-p\right)\right)p^{2}}{n{\left(1-p\right)}^{2}}u^{p}\sum_{a=1}^{n}\sum_{b=1}^{n}\left[\left(\frac{\partial^{2}}{\partial^{2}x_{a,t}}u^{p-1}\right)\left(\frac{\partial^{2}}{\partial^{2}x_{b,t}}u^{p-1}\right)\right]\\ \; & +\frac{p}{1-p}\sum_{a=1}^{n}\left[\left(p-1\right)u^{p-2}\left(\frac{\partial^{2}}{\partial^{2}x_{a,t}}u^{p}\right)\sum_{b=1}^{n}\left(\frac{\partial^{2}}{\partial^{2}x_{b,t}}u^{p}\right)+pu^{p-1}\frac{\partial^{2}}{\partial^{2}x_{a,t}}\left(u^{p-1}\sum_{b=1}^{n}\left(\frac{\partial^{2}}{\partial^{2}x_{b,t}}u^{p}\right)\right)\right]\\ \; & =\frac{\left(\mu -n\left(1-p\right)\right)p^{2}}{n{\left(1-p\right)}^{2}}u^{p}\sum_{a=1}^{n}\sum_{b=1}^{n}\left[\left(\frac{\partial^{2}}{\partial^{2}x_{a,t}}u^{p-1}\right)\left(\frac{\partial^{2}}{\partial^{2}x_{b,t}}u^{p-1}\right)\right]\\ \; & +\frac{p}{1-p}\sum_{a=1}^{n}\sum_{b=1}^{n}\left[\left(p-1\right)u^{p-2}\left(\frac{\partial^{2}}{\partial^{2}x_{a,t}}u^{p}\right)\left(\frac{\partial^{2}}{\partial^{2}x_{b,t}}u^{p}\right)\right.\\ \; & \left.+pu^{p-1}\frac{\partial^{2}}{\partial^{2}x_{a,t}}\left(u^{p-1}\left(\frac{\partial^{2}}{\partial^{2}x_{b,t}}u^{p}\right)\right)\right]\\ \; & =\sum_{a=1}^{n}\sum_{b=1}^{n}T_{a,b}, \end{array}$$

where

(25) $$\begin{array}{cc} T_{a,b} & =\frac{\left(\mu -n\left(1-p\right)\right)p^{2}}{n{\left(1-p\right)}^{2}}u^{p}\left[\left(\frac{\partial^{2}}{\partial^{2}x_{a,t}}u^{p-1}\right)\left(\frac{\partial^{2}}{\partial^{2}x_{b,t}}u^{p-1}\right)\right]\\ & +\frac{p}{1-p}\left[\left(p-1\right)u^{p-2}\left(\frac{\partial^{2}}{\partial^{2}x_{a,t}}u^{p}\right)\left(\frac{\partial^{2}}{\partial^{2}x_{b,t}}u^{p}\right)\right.\\ \; & \left.+pu^{p-1}\frac{\partial^{2}}{\partial^{2}x_{a,t}}\left(u^{p-1}\left(\frac{\partial^{2}}{\partial^{2}x_{b,t}}u^{p}\right)\right)\right]. \end{array}$$

For convenience, introduce the notation $u_{i,j}:=\frac{\partial^{i+j}u}{\partial^{i}x_{a,t}\partial^{j}x_{b,t}}$ . Then, by calculating the differentiation formulas in (25) and substituting $\frac{\partial^{i+j}u}{\partial^{i}x_{a,t}\partial^{j}x_{b,t}}=u_{i,j}$ , we have $T_{a,b}=-\frac{u^{3p-6}p^{2}}{(p-1)n}T_{a,b}$ , where

(26) $$\begin{array}{cc} T_{a,b} & =4np^{4}u_{0,1}^{2}u_{1,0}^{2}+2np^{3}uu_{0,1}^{2}u_{2,0}+8np^{3}uu_{0,1}u_{1,0}u_{1,1}\\ & +4np^{3}uu_{0,2}u_{1,0}^{2}-15np^{3}u_{0,1}^{2}u_{1,0}^{2}-\mu p^{3}u_{0,1}^{2}u_{1,0}^{2}+2np^{2}u^{2}u_{0,1}u_{2,1}\\ & +2np^{2}u^{2}u_{0,2}u_{2,0}+4np^{2}u^{2}u_{1,0}u_{1,2}+2np^{2}u^{2}u_{1,1}^{2}-3np^{2}uu_{0,1}^{2}u_{2,0}\\ & -20np^{2}uu_{0,1}u_{1,0}u_{1,1}-8np^{2}uu_{0,2}u_{1,0}^{2}-\mu p^{2}uu_{0,1}^{2}u_{2,0}-\mu p^{2}uu_{0,2}u_{1,0}^{2}\\ & +16np^{2}u_{0,1}^{2}u_{1,0}^{2}+5\mu p^{2}u_{0,1}^{2}u_{1,0}^{2}+npu^{3}u_{2,2}-2npu^{2}u_{0,1}u_{2,1}\\ & -npu^{2}u_{0,2}u_{2,0}-4npu^{2}u_{1,0}u_{1,2}-2npu^{2}u_{1,1}^{2}-\mu pu^{2}u_{0,2}u_{2,0}\\ & -npuu_{0,1}^{2}u_{2,0}+12npuu_{0,1}u_{1,0}u_{1,1}+2npuu_{0,2}u_{1,0}^{2}+3\mu puu_{0,1}^{2}u_{2,0}\\ & +3\mu puu_{0,2}u_{1,0}^{2}-npu_{0,1}^{2}u_{1,0}^{2}-8\mu pu_{0,1}^{2}u_{1,0}^{2}-nu^{2}u_{0,2}u_{2,0}\\ & +\mu u^{2}u_{0,2}u_{2,0}+2nuu_{0,1}^{2}u_{2,0}+2nuu_{0,2}u_{1,0}^{2}-2\mu uu_{0,1}^{2}u_{2,0}\\ & -2\mu uu_{0,2}u_{1,0}^{2}-4nu_{0,1}^{2}u_{1,0}^{2}+4\mu u_{0,1}^{2}u_{1,0}^{2}, \end{array}$$

which is a fourth-order differential form.

From (22)–(25), we have

(27) $$\frac{d^{2}}{d^{2}t}N_{p}\left(u\right)\leq -\frac{p^{2}\mu }{n^{2}}e^{\frac{\mu }{n}H_{p}\left(u\right)}\frac{1}{\int u^{p}dx_{t}}\int u^{3p-6}E_{2,n}dx_{t},$$

where $E_{2,n}=\sum_{a=1}^{n}\sum_{b=1}^{n}\frac{T_{a,b}}{p-1}$ and $T_{a,b}$ is defined in (26). Then, the problem $\frac{d^{2}}{d^{2}t}N_{p}\left(u\right)\leq 0$ can be transformed to $\int u^{3p-6}E_{2,n}dx_{t}\geq 0$ . Thus, Lemma 1 is proved.

## 3. A Generalized Version of CREP

In this section, we prove a generalized CREP using the procedure given in Section 2.

**Theorem** **2.** *Let $u(x_{t})$ be a probability density in $R^{n}$ solving (6) and satisfying (14). Then, we give a propositional formula Φ(n,p,μ) such that the p-th Rényi entropy power defined in (2) satisfies*

(28) $$\begin{array}{c} \frac{d^{2}}{d^{2}t}N_{p}\left(x_{t}\right)\leq 0, \end{array}$$

*under the condition Φ(n,p,μ) , that is $N_{p}\left(x_{t}\right)$ is concave under Φ(n,p,μ) .*

The proof of the above theorem consists of three steps, which are given in the following three subsections.

### 3.1. Reduce to a Finite Problem

We first give an inequality constraint. Denote $|\nabla^{2}{f|}^{2}=\sum_{i,j}{\left(\frac{\partial^{2}f}{\partial x_{i}\partial x_{j}}\right)}^{2}$ . Then, based on the trace inequality $|\nabla^{2}{f|}^{2}\geq \frac{1}{n}{\left(\Delta f\right)}^{2}$ , we give an inequality constraint:

(29) $$\begin{array}{c} I_{1}=\frac{u^{p}}{u^{3p-6}}\left[|\nabla^{2}u^{p-1}{|}^{2}-\frac{1}{n}{\left(\Delta u^{p-1}\right)}^{2}\right]=\sum_{a=1}^{n}\sum_{b=1}^{n}I_{1,a,b}\geq 0, \end{array}$$

where $I_{1,a,b}=u^{6-2p}\left[{\left(\frac{\partial^{2}u^{p-1}}{\partial x_{a,t}\partial x_{b,t}}\right)}^{2}-\frac{1}{n}\frac{\partial^{2}u^{p-1}}{\partial^{2}x_{a,t}}\frac{\partial^{2}u^{p-1}}{\partial^{2}x_{b,t}}\right]$ .

From (27) and (29), in order for (28) to be true, it suffices to solve

**Problem** **1.** *Find a formula Φ(n,p,μ) such that*

$$\begin{array}{c} E_{2,n}\geq {\tilde{E}}_{2,n}=E_{2,n}+c_{1}I_{1}=\sum_{a=1}^{n}\sum_{b=1}^{n}\left(\frac{1}{p-1}T_{a,b}+c_{1}I_{1,a,b}\right)\geq 0, \end{array}$$

*under the conditions $c_{1}\leq 0$ , $p\geq 1-\frac{\mu }{n}$ , $R_{i,a,b}=0,i=1,\ldots ,28$ given in (15).*

Since $\sum_{a=1}^{n}\sum_{b=1}^{n}T_{a,b}=\sum_{a=1}^{n}\sum_{b=1}^{n}T_{b,a}$ and $I_{1,a,b}=R_{1,b,a}^{\left(I\right)}$ , we have

(30) $$\begin{array}{cc} {\tilde{E}}_{2,n} & =\frac{1}{2}\sum_{a=1}^{n}\sum_{b=1}^{n}\left[\frac{1}{p-1}\left(T_{a,b}+T_{b,a}\right)+c_{1}\left(I_{1,a,b}+I_{1,b,a}\right)\right]=\frac{1}{2}\sum_{a=1}^{n}\sum_{b=1}^{n}L_{a,b}, \end{array}$$

where $L_{a,b}=\frac{1}{p-1}\left(T_{a,b}+T_{b,a}\right)+c_{1}\left(I_{1,a,b}+I_{1,b,a}\right)$ .

From (30), in order to solve Problem 1, it suffices to solve

**Problem** **2.** *Find a formula Φ(n,p,μ) such that $L_{a,b}\geq 0$ under the conditions $c_{1}\leq 0$ , $p\geq 1-\frac{\mu }{n}$ , and $R_{i,a,b}=0,i=1,\ldots ,28$ .*

### 3.2. Simplify the Problem with the Equational Constraints

In this section, we simplify $L_{a,b}$ in Problem 2 with the equational constraints $C_{2,n}$ in (15). Note that the subscripts *a* and *b* are fixed and are treated as symbols.

Our goal is to reduce $L_{a,b}$ into a quadratic form in certain new variables. The new variables are all the monomials in $R[V_{a,b}]$ with degree 2 and total order 2:

$$\begin{array}{cc} & m_{1}=uu_{0,2},\;m_{2}=uu_{1,1},\;m_{3}=uu_{2,0},\\ & m_{4}=u_{0,1}^{2},\;m_{5}=u_{1,0}u_{0,1},\;m_{6}=u_{1,0}^{2}, \end{array}$$

where $V_{a,b}$ is defined in (8).

We simplify the constraints in (15) as follows. A quadratic monomial in $m_{i}$ is called a *quadratic monomial*. Write monomials in $C_{2,n}=\left\{R_{i},i=1,\ldots ,N_{1}\right\}$ as quadratic monomials if possible. Performing Gaussian elimination to $C_{2,n}$ by treating the monomials as variables, and according to a monomial order such that a quadratic monomial is less than a non-quadratic monomial, we obtain

$${\tilde{C}}_{2,n}=C_{2,n,1}\cup C_{2,n,2},$$

where $C_{2,n,1}$ is the set of quadratic forms in $m_{i}$ , $C_{2,n,2}$ is the set of non-quadratic forms, and ${\mathrm{Span}}_{R}\left(C_{2,n}\right)={\mathrm{Span}}_{R}\left({\tilde{C}}_{2,n}\right)$ . We obtain $C_{2,n,1}=\left\{{\hat{R}}_{i},i=1,\ldots ,9\right\}$ and $C_{2,n,2}=\left\{{\tilde{R}}_{i},i=1,\ldots ,13\right\}$ , where

$$\begin{array}{cc} {\hat{R}}_{1}=2m_{1}m_{5}+\frac{2(3p-5)}{3}m_{4}m_{5},\; & {\hat{R}}_{2}=m_{2}m_{6}+\frac{3p-5}{3}m_{5}m_{6},\\ {\hat{R}}_{3}=-6m_{3}m_{5}+2\left(5-3p\right)m_{5}m_{6},\; & {\hat{R}}_{4}=\left(3p-5\right)m_{4}^{2}+3m_{1}m_{4},\\ {\hat{R}}_{5}=\left(3p-5\right)m_{6}^{2}+3m_{3}m_{6},\; & {\hat{R}}_{6}=\left(3p-5\right)m_{4}m_{5}+3m_{2}m_{4},\\ {\hat{R}}_{7}=\left(3p-5\right)m_{5}^{2}+2m_{2}m_{5}+m_{3}m_{4}, & \\ {\hat{R}}_{8}=m_{1}m_{3}-m_{2}^{2}+\frac{9p-12}{2}m_{3}m_{4}+\frac{9p^{2}-27p+20}{2}m_{5}^{2}, & \\ {\hat{R}}_{9}=m_{1}m_{6}-m_{3}m_{4}.* \end{array}$$

$$\begin{array}{c} {\tilde{R}}_{1}=u^{3}u_{0,4}+\left(3-3p\right)m_{1}^{2}+\left(9p^{3}-36p^{2}+47p-20\right)m_{4}^{2},\\ {\tilde{R}}_{2}=u^{3}u_{1,3}+\left(3-3p\right)m_{1}m_{2}+\left(9p^{3}-36p^{2}+47p-20\right)m_{4}m_{5},\\ {\tilde{R}}_{3}=u^{3}u_{3,1}+\left(3-3p\right)m_{2}m_{3}+\left(-9p^{2}+21p-12\right)m_{3}m_{5},\\ {\tilde{R}}_{4}=u^{3}u_{4,0}+\left(3-3p\right)m_{3}^{2}+\left(9p^{3}-36p^{2}+47p-20\right)m_{6}^{2},\\ {\tilde{R}}_{5}=u^{2}u_{0,1}u_{0,3}+m_{1}^{2}+\frac{-9p^{2}+27p-20}{3}m_{4}^{2},\\ {\tilde{R}}_{6}=u^{2}u_{0,1}u_{1,2}+m_{1}m_{2}+\frac{-9p^{2}+27p-20}{3}m_{4}m_{5},\\ {\tilde{R}}_{7}=u^{2}u_{0,1}u_{3,0}+m_{2}m_{3}+\frac{-9p^{2}+27p-20}{3}m_{5}m_{6},\\ {\tilde{R}}_{8}=u^{2}u_{1,0}u_{0,3}+m_{1}m_{2}+\frac{-9p^{2}+27p-20}{3}m_{4}m_{5},\\ {\tilde{R}}_{9}=u^{2}u_{1,0}u_{2,1}+m_{2}m_{3}+\frac{-9p^{2}+27p-20}{3}m_{5}m_{6},\\ {\tilde{R}}_{10}=u^{2}u_{1,0}u_{3,0}+m_{3}^{2}+\frac{-9p^{2}+27p-20}{3}m_{6}^{2},\\ {\tilde{R}}_{11}=u^{3}u_{2,2}+\left(3-3p\right)m_{2}^{2}+\frac{9p^{2}-21p+12}{2}m_{3}m_{4}+\frac{27p^{3}-108p^{2}+141p-60}{2}m_{5}^{2},\\ {\tilde{R}}_{12}=u^{2}u_{0,1}u_{2,1}+m_{2}^{2}+\frac{4-3p}{2}m_{3}m_{4}+\frac{-9p^{2}+27p-20}{2}m_{5}^{2},\\ {\tilde{R}}_{13}=u^{2}u_{1,0}u_{1,2}+m_{2}^{2}+\frac{4-3p}{2}m_{3}m_{4}+\frac{-9p^{2}+27p-20}{2}m_{5}^{2}. \end{array}$$

We now simplify $L_{a,b}$ using $C_{2,n,1}$ and $C_{2,n,2}$ . Eliminating the non-quadratic monomials in $L_{a,b}$ using $C_{2,n,2}$ , and performing further reduction by $C_{2,n,1}$ , we have

(31) $$\begin{array}{cc} \; & {\hat{L}}_{a,b}=L_{a,b}-2\left(p^{3}c_{1}+4np^{2}-4p^{2}c_{1}-6np+5pc_{1}-2c_{1}\right){\hat{R}}_{7}\\ \; & -\frac{2}{n}\left(2n^{2}p-p^{2}c_{1}+n^{2}-n\mu +2pc_{1}-c_{1}\right){\hat{R}}_{8}\\ \; & -\frac{1}{n}\left(6n^{2}p^{2}-2p^{3}c_{1}-5n^{2}p-2np\mu +8p^{2}c_{1}-4n^{2}+4n\mu -10pc_{1}+4c_{1}\right){\hat{R}}_{9}\\ \; & -\frac{2np}{p-1}{\tilde{R}}_{11}-6np{\tilde{R}}_{12}-6np{\tilde{R}}_{13}\\ \; & =\left(2np+2n-2\mu \right)m_{2}^{2}+\left(5np-5np^{2}+5p\mu +4n-4\mu \right)m_{3}m_{4}\\ \; & +\left(18np^{2}-7np^{3}+7p^{2}\mu -3np-19p\mu -12n+12\mu \right)m_{5}^{2}\\ \; & +\frac{c_{1}}{n}[\left(2n-2\right)\left(p^{2}-2p+1\right)m_{2}^{2}+\left(4n-2np+5p-4\right)\left(p^{2}-2p+1\right)m_{3}m_{4}\\ \; & +\left(14np-4np^{2}+7p^{2}-12n-19p+12\right)\left(p^{2}-2p+1\right)m_{5}^{2}]. \end{array}$$

In order for ${\hat{L}}_{a,b}\geq 0$ to be true, we need to eliminate the monomial $m_{3}m_{4}$ from ${\hat{L}}_{a,b}$ , which can be done with ${\hat{R}}_{7}$ as follows.

(32) $${\hat{L}}_{a,b}+p_{7}{\hat{R}}_{7}=A_{1}m_{2}^{2}+A_{2}m_{2}m_{5}+A_{3}m_{5}^{2},$$

where

$$\begin{array}{cc} \; & p_{7}=(2np^{3}c_{1}+5n^{2}p^{2}-8np^{2}c_{1}-5p^{3}c_{1}-5n^{2}p-5np\mu +10npc_{1}\\ \; & \;+14p^{2}c_{1}-4n^{2}+4n\mu -4nc_{1}-13pc_{1}+4c_{1})/n,\\ \; & A_{1}=-2c_{1}p^{2}/n+4c_{1}p/n+2np+2c_{1}+2c_{1}p^{2}-4c_{1}p-2c_{1}/n-2\mu +2n,\\ \; & A_{2}=4c_{1}p^{3}-16c_{1}p^{2}-10c_{1}p^{3}/n-10p\mu +20c_{1}p+28c_{1}p^{2}/n-26c_{1}p/n\\ \; & \;+10np^{2}-10np+8\mu -8c_{1}+8c_{1}/n-8n,\\ \; & A_{3}=-8\mu +8n+26c_{1}p^{2}-24c_{1}p-8c_{1}/n-12c_{1}p^{3}+2c_{1}p^{4}+8c_{1}-52c_{1}p^{2}/n\\ \; & \;+34c_{1}p/n+34c_{1}p^{3}/n-8c_{1}p^{4}/n+18p\mu +8np^{3}-22np^{2}-8p^{2}\mu +10np. \end{array}$$

### 3.3. Compute Φ(n,p,μ)

From (32), in order to solve Problem 2, it suffices to solve

**Problem** **3.** *Find a propositional formula Φ(n,p,μ) such that*

(33) $$\Phi \left(n,p,\mu \right)\Leftrightarrow \exists c_{1}\left(c_{1}\leq 0\wedge p\geq 1-\frac{\mu }{n}\wedge A_{1}m_{2}^{2}+A_{2}m_{2}m_{5}+A_{3}m_{5}^{2}\geq 0\right).$$

In principle, Problem 3 can be solved with the quantifier elimination [24]. In this paper, the problem is special, and an explicit proof is given.

By the knowledge of linear algebra, $A_{1}m_{2}^{2}+A_{2}m_{2}m_{5}+A_{3}m_{5}^{2}\geq 0$ is equivalent to $\Delta_{1}=A_{1}=\frac{2}{n}s_{1}\geq 0$ , $\Delta_{2}=A_{3}=\frac{2}{n}s_{2}\geq 0$ , $\Delta_{3}=A_{1}A_{3}-\frac{1}{4}A_{2}^{2}=\frac{p}{n^{2}}s_{3}\geq 0$ , where

$$\begin{array}{cc} \; & s_{1}={\left(p-1\right)}^{2}\left(n-1\right)c_{1}+n^{2}\left(p+1\right)-n\mu ,\\ \; & s_{2}={\left(p-1\right)}^{2}\left(n{\left(p-2\right)}^{2}-4p^{2}+9p-4\right)c_{1}\\ \; & +n^{2}\left(4p^{3}-11p^{2}+5p+4\right)-\left(4p^{2}-9p+4\right)n\mu ,\\ \; & s_{3}=\left(4-9p\right)n^{2}\left(\mu -\mu_{3}\right)\left(\mu -\mu_{4}\right), \end{array}$$

and $\mu_{3}$ and $\mu_{4}$ are defined in (37). Furthermore, $p\neq \frac{4}{9}$ is assumed in $\mu_{4}$ . Thus, the following lemma is proved.

**Lemma** **5.** *We have*

(34) $$\Phi \left(n,p,\mu \right)\Leftrightarrow \exists c_{1}\left(c_{1}\leq 0,\;s_{1}\geq 0,\;s_{2}\geq 0,\;s_{3}\geq 0,\;p\geq 1-\mu /n\right).$$

We present an explicit formula for Φ in (34). First, we introduce the following parameters.

(35) $$\begin{array}{ccc} n_{1}=\frac{9-\sqrt{17}}{8},\; & n_{2}=\frac{9+\sqrt{17}}{8},\; & n_{3}=\left(\sqrt{17}+1\right)/2,\\ \theta_{1}=-\frac{2n}{{\left(p-1\right)}^{2}},\; & \theta_{2}=\frac{2n^{2}p\left(9p-13\right)}{{\left(p-1\right)}^{2}\left(4n+9p-4\right)},\; & \theta_{3}=\left(\sqrt{17}-9\right)n,\\ \theta_{4}=\frac{n^{2}p-n^{2}-n\mu }{{\left(p-1\right)}^{2}},\; & \theta_{5}=\frac{n^{2}\left(9p^{2}-13p-4\right)-n\left(9p-4\right)\mu }{{\left(p-1\right)}^{2}\left(4n+9p-4\right)},\; & \theta_{6}=\frac{-4n\left(\sqrt{17}-1\right)\mu +8n^{2}}{\sqrt{17}+1},\\ \theta_{7}=n\left(1-p\right),\; & \theta_{8}=5n/9,\; & \theta_{9}=-\frac{162n}{25},\\ \theta_{10}=\frac{64n}{\sqrt{17}-9},\; & \theta_{11}=\frac{8\left(9\sqrt{17}+23\right)n^{2}}{-32n-49+9\sqrt{17}},\; & \theta_{12}=-\frac{16\left(11\sqrt{17}+47\right)n\mu +152n^{2}}{26\sqrt{17}n+73\sqrt{17}+118n+305},\\ \theta_{13}=-\frac{4n(\mu \sqrt{17}+2n+\mu )}{\sqrt{17}-1},\; & \theta_{14}=-\frac{8n(22\mu \sqrt{17}-19n-94\mu )}{26\sqrt{17}n+73\sqrt{17}-118n-305},\; & \theta_{15}=-\frac{9}{5}n^{2}-\frac{81}{25}\mu n,\\ \theta_{16}=\left(\sqrt{17}-1\right)n/8,\; & \phi_{1}≜p\geq 1-\frac{1}{n},\; & \phi_{2}≜\mu =2+n\left(p-1\right),\\ \phi_{3}≜p>1-\frac{1}{n}. \end{array}$$

We define Φ in (34) using Table 1, where * means *⌀*. Define T(i,j) to be the formula in the *i*-th row and the *j*-th column in Table 1. Then, we denote

(36) T(i,j)≜T(i,1)∧T(i,j)fori=1,…,8,j=2,3,4.

For example, T(1,2) is $p>n_{2}\wedge \theta_{4}>\theta_{1}\wedge \theta_{5}\leq 0$ , which means that if p,n,μ satisfy T(1,2) , then there exists a $c_{1}\leq 0$ such that (33) is true and the CREP is valid. T(1,3)=⌀ , which means that there exist no values for p,n,μ such that (33), and the CREP is true in this case.

We now give the main result of the paper, which implies Theorem 2. The proof for the theorem can be found in Section 3.5.

**Theorem** **3.** *The sufficient and necessary condition for Problem 3— that is, (33) must be true—is*

$$\Phi \left(n,p,\mu \right)=\vee_{i=1}^{8}\vee_{j=2}^{4}T\left(i,j\right),$$

*where T(i,j) is defined in (36) and ∨ means disjunction.*

### 3.4. Compare with Existing Results

We show that our result includes the result proved in [22] and more essential results.

In [22], the CREP was proved under the conditions μ=2+n(p−1) and $p\geq 1-\frac{1}{n}$ . Obviously, the result proved in [22] is a special case of T(i,4),i=1,…,8 in Table 1.

We can also prove the result in [22] directly as follows. Set μ=2+n(p−1) and $c_{1}=-\frac{2n}{{\left(p-1\right)}^{2}}\leq 0$ in (31), we obtain ${\hat{L}}_{a,b}=0$ . In addition, the condition $p\geq 1-\frac{\mu }{n}$ implies $p\geq 1-\frac{1}{n}$ . So, when μ=2+n(p−1) and $p\geq 1-\frac{1}{n}$ , the CREP is proved based on our proof procedure.

We can use the SDP code in ([15], Appendix B) to verify the result in Table 1 for given values of μ,p,n . For instance, for $\mu =2,p=\frac{11}{5},n=2$ , the condition $p\geq 1-\frac{\mu }{n}$ is satisfied naturally. With the SDP code in [15], we obtain ${\hat{L}}_{a,b}+\frac{172}{25}{\hat{R}}_{7}={\left(2\sqrt{2}m_{2}+\frac{344}{100\sqrt{2}}m_{5}\right)}^{2}+\frac{22}{625}m_{5}^{2}\geq 0$ with $c_{1}=-\frac{5}{9}$ . Thus, the CREP is proved when $\mu =2,p=\frac{11}{5},n=2$ . This case $\left[\mu =2,p=\frac{11}{5},n=2,c_{1}=-\frac{5}{9}\right]$ is included in T(1,2) in Table 1. Note that μ=2+n(p−1) is not satisfied for these parameters, and thus our condition Φ(n,p,μ) is strictly larger than those given in [22]. More precisely, the points (n,p,μ) satisfying the conditions $\mu =2+n\left(p-1\right),p\geq 1-\frac{1}{n}$ given in [22] consist of a two-dimensional subset of $R^{3}$ , while the points satisfying the condition Φ(n,p,μ) consist of a three-dimensional subset of $R^{3}$ , as shown by the following result.

**Property** **2.** *The points satisfying the condition Φ(n,p,μ) consist of a three-dimensional subset of $R^{3}$ .*

**Proof.**  We show that the points satisfying T(1,2) consist of a three-dimensional subset of $R^{3}$ .

From Table 1, we have $T\left(1,2\right)=\left[F_{1}>0\wedge F_{2}>0\wedge F_{3}\leq 0\right]$ , where $F_{1}=p-\frac{9+\sqrt{17}}{8}$ , $F_{2}=\frac{n^{2}p-n^{2}-n\mu }{{\left(p-1\right)}^{2}}+\frac{2n}{{\left(p-1\right)}^{2}}$ , $F_{3}=\frac{n^{2}\left(9p^{2}-13p-4\right)-n\left(9p-4\right)\mu }{{\left(p-1\right)}^{2}\left(4n+9p-4\right)}$ . Under the condition $F_{1}>0$ , we can reduce the inequality $F_{2}>0$ to the form μ<2+n(p−1) and reduce the inequality $F_{3}\leq 0$ to the form $\mu \geq \frac{n(9p^{2}-13p-4)}{9p-4}$ . Thus, $T\left(1,2\right)=[p>\frac{9+\sqrt{17}}{8}\wedge \frac{n(9p^{2}-13p-4)}{9p-4}\leq \mu <n\left(p-1\right)+2]$ . Since $n\left(p-1\right)+2-\frac{n(9p^{2}-13p-4)}{9p-4}=\frac{8n+2(9p-4)}{9p-4}>0$ under the condition $p>\frac{9+\sqrt{17}}{8}$ , T(1,2) defines a three-dimensional subset of $R^{3}$ . □

### 3.5. Proof of Theorem 3

In order to make the proof precise, we introduce the following parameters:

(37) $$\begin{array}{cc} \mu_{1} & =({\left(p-1\right)}^{2}\left(n-1\right)c_{1}+n^{2}\left(p+1\right))/n,\\ \mu_{2} & {=(\left(p-1\right)}^{2}\left(n{\left(p-2\right)}^{2}-4p^{2}+9p-4\right)c_{1}+n^{2}(4p^{3}-11p^{2}+5p\\ \; & +4))/\left(n\left(4p^{2}-9p+4\right)\right)\\ \mu_{3} & =(n^{2}p-p^{2}c_{1}-n^{2}+2pc_{1}-c_{1})/n,\\ \mu_{4} & =(9n^{2}p^{2}-4np^{2}c_{1}-9p^{3}c_{1}-13n^{2}p+8npc_{1}+22p^{2}c_{1}-4n^{2}-4nc_{1}-17pc_{1}\\ \; & +4c_{1})/\left(n\left(9p-4\right)\right),\\ \mu_{5} & =-\left(nc_{1}\sqrt{17}-c_{1}\sqrt{17}+136n^{2}+17nc_{1}-17c_{1}\right)/\left(4n\left(\sqrt{17}-17\right)\right),\\ \mu_{6} & =-\left(c_{1}\sqrt{17}-8n^{2}+c_{1}\right)/\left(4n\left(\sqrt{17}-1\right)\right),\\ \mu_{7} & =-\left(26nc_{1}\sqrt{17}+73c_{1}\sqrt{17}+152n^{2}+118nc_{1}+305c_{1}\right)/\left(16n\left(11\sqrt{17}+47\right)\right),\\ \mu_{8} & =-\left(nc_{1}\sqrt{17}-c_{1}\sqrt{17}-136n^{2}-17nc_{1}+17c_{1}\right)/\left(4n\left(\sqrt{17}+17\right)\right),\\ \mu_{9} & =-\left(c_{1}\sqrt{17}+8n^{2}-c_{1}\right)/\left(4n\left(\sqrt{17}+1\right)\right),\\ \mu_{10} & =-\left(26nc_{1}\sqrt{17}+73c_{1}\sqrt{17}-152n^{2}-118nc_{1}-305c_{1}\right)/\left(16n\left(11\sqrt{17}-47\right)\right),\\ \mu_{11} & =\left(117n^{2}+25nc_{1}-25c_{1}\right)/\left(81n\right),\\ \mu_{12} & =\left(7218n^{2}+1225nc_{1}-400c_{1}\right)/\left(1296n\right),\\ \mu_{13} & =-\left(5\left(9n^{2}+5c_{1}\right)\right)/\left(81n\right),\\ \eta_{1} & =\frac{2n^{2}}{p-1},\eta_{2}=-\frac{16n^{2}}{\sqrt{17}-1},\eta_{3}=-\frac{18}{5}n^{2}. \end{array}$$

We first treat the three inequalities $s_{1}\geq 0$ , $s_{2}\geq 0$ , $s_{3}\geq 0$ . Firstly, $s_{1}\geq 0$ is equivalent to $\mu \leq \mu_{1}$ . Secondly, since the roots of $4p^{2}-9p+4=0$ are $n_{1}$ and $n_{2}$ , we have $s_{2}\geq 0\Leftrightarrow \mu \leq \mu_{2}$ if $p<n_{1}$ or $p>n_{2}$ ; and $s_{2}\geq 0\Leftrightarrow \mu \geq \mu_{2}$ if $n_{1}<p<n_{2}$ . In order to analyze $s_{3}\geq 0$ , we first compute

(38) $$\mu_{3}-\mu_{4}=\frac{4({\left(p-1\right)}^{2}c_{1}+2n)}{9p-4}.$$

Therefore, $s_{3}\geq 0$ can be divided into four cases: $s_{3}\geq 0\Leftrightarrow \mu_{4}\leq \mu \leq \mu_{3}$ if $p>\frac{4}{9}$ and $\theta_{1}<c_{1}$ ; $s_{3}\geq 0\Leftrightarrow \mu_{3}\leq \mu \leq \mu_{4}$ if $p>\frac{4}{9}$ and $c_{1}<\theta_{1}$ ; $s_{3}\geq 0\Leftrightarrow \mu \geq \mu_{3}\;\mathrm{or}\;\mu \leq \mu_{4}$ if $p<\frac{4}{9}$ and $c_{1}<\theta_{1}$ ; $s_{3}\geq 0\Leftrightarrow \mu \geq \mu_{4}\;\mathrm{or}\;\mu \leq \mu_{3}$ if $p<\frac{4}{9}$ and $\theta_{1}<c_{1}$ . Finally, $p\geq 1-\frac{\mu }{n}$ is equivalent to $\mu \geq \theta_{7}$ .

Based on the above analysis and (34), Φ(n,p,μ) can be divided into six cases:

(39) $$\begin{array}{cc} \Phi (n,p,\mu ) & \Leftrightarrow \max\left(\mu_{4},\theta_{7}\right)\leq \mu \leq \min\left(\mu_{1},\mu_{2},\mu_{3}\right),\;\mathrm{if}\;\left(p\in \left(\frac{4}{9},n_{1}\right)\;\mathrm{or}\;p>n_{2}\right)\\ & \;\mathrm{and}\;\theta_{1}<c_{1}\leq 0;\\ \Phi (n,p,\mu ) & \Leftrightarrow \max\left(\mu_{2},\mu_{4},\theta_{7}\right)\leq \mu \leq \min\left(\mu_{1},\mu_{3}\right),\;\mathrm{if}\;p\in \left(n_{1},n_{2}\right)\\ & \;\mathrm{or}\;\theta_{1}<c_{1}\leq 0;\\ \Phi \left(n,p,\mu \right) & \Leftrightarrow \max\left(\mu_{3},\theta_{7}\right)\leq \mu \leq \min\left(\mu_{1},\mu_{2},\mu_{4}\right),\;\mathrm{if}\;\left(p\in \left(\frac{4}{9},n_{1}\right)\mathrm{or}p>n_{2}\right)\\ & \mathrm{or}c_{1}<\theta_{1};\\ \Phi \left(n,p,\mu \right) & \Leftrightarrow \max\left(\mu_{2},\mu_{3},\theta_{7}\right)\leq \mu \leq \min(\mu_{1},\mu_{4}),\mathrm{if}p\in (n_{1},n_{2})\mathrm{or}c_{1}<\theta_{1};\\ \Phi \left(n,p,\mu \right) & \Leftrightarrow \theta_{7}\leq \mu \leq \min\left(\mu_{1},\mu_{2},\mu_{4}\right)\;\mathrm{or}\;\max\left(\mu_{3},\theta_{7}\right)\leq \mu \leq \min\left(\mu_{1},\mu_{2}\right),\\ & \mathrm{if}p<\frac{4}{9}\mathrm{or}c_{1}<\theta_{1};\\ \Phi \left(n,p,\mu \right) & \Leftrightarrow \theta_{7}\leq \mu \leq \min\left(\mu_{1},\mu_{2},\mu_{3}\right)\;\mathrm{or}\;\max\left(\mu_{4},\theta_{7}\right)\leq \mu \leq \min\left(\mu_{1},\mu_{2}\right),\\ & \mathrm{if}p<\frac{4}{9}\;\mathrm{or}\;\theta_{1}<c_{1}\leq 0. \end{array}$$

The special cases $p=\frac{4}{9},n_{1},n_{2}$ , and $c=\theta_{1}$ need to be considered differently.

Below, we give a detailed analysis of the above six cases, which leads to the results in Table 1. We first have the following formulas:

(40) $$\begin{array}{ccc} \mu_{1}-\mu_{3} & = & {\left(p-1\right)}^{2}c_{1}+2n, \end{array}$$

(41) $$\begin{array}{ccc} \mu_{1}-\mu_{4} & = & \frac{9p({\left(p-1\right)}^{2}c_{1}+2n)}{9p-4}, \end{array}$$

(42) $$\begin{array}{ccc} \mu_{2}-\mu_{3} & = & \frac{2{\left(p-2\right)}^{2}\left(\left(1/2\right){\left(p-1\right)}^{2}c_{1}+n\right)}{\left(4p^{2}-9p+4\right)}, \end{array}$$

(43) $$\begin{array}{ccc} \mu_{2}-\mu_{4} & = & \frac{p{\left(3p-4\right)}^{2}\left({\left(p-1\right)}^{2}c_{1}+2n\right)}{\left(4p^{2}-9p+4\right)\left(9p-4\right)}, \end{array}$$

(44) $$\begin{array}{ccc} \mu_{4}-\theta_{7} & = & \frac{-{\left(p-1\right)}^{2}\left(4n+9p-4\right)c_{1}+2n^{2}p\left(9p-13\right)}{n(9p-4)}, \end{array}$$

(45) $$\begin{array}{ccc} \mu_{3}-\theta_{7} & = & \frac{\left(p-1\right)\left(2n^{2}-pc_{1}+c_{1}\right)}{n}, \end{array}$$

(46) $$\begin{array}{ccc} \theta_{2}-\theta_{1} & = & \frac{18n\left(np-n+1\right)\left(p-\frac{4}{9}\right)}{{\left(p-1\right)}^{2}\left(4n+9p-4\right)}, \end{array}$$

(47) $$\begin{array}{ccc} \theta_{1}-\eta_{1} & = & -\frac{2n(np-n+1)}{{\left(p-1\right)}^{2}}, \end{array}$$

(48) $$\begin{array}{ccc} \eta_{1}-\theta_{2} & = & \frac{8n^{2}\left(np-n+1\right)}{{\left(p-1\right)}^{2}\left(4n+9p-4\right)}. \end{array}$$

Firstly, we have the following formulas which eliminate $c_{1}$ .

(49) $$\begin{array}{cc} \mu \leq \mu_{3}\Leftrightarrow c_{1}\leq \theta_{4},\; & \mu \geq \mu_{4}\Leftrightarrow c_{1}\geq \theta_{5},\;\mathrm{if}\;p>\frac{4}{9},\\ \mu \geq \mu_{4}\Leftrightarrow c_{1}\leq \theta_{5},\;\mathrm{if}\;p<\frac{4}{9},\; & \mu \leq \mu_{4}\Leftrightarrow c_{1}\leq \theta_{5},\;\mathrm{if}\;p>\frac{4}{9},\\ \mu \leq \mu_{4}\Leftrightarrow c_{1}\geq \theta_{5},\;\mathrm{if}\;p<\frac{4}{9},\; & \mu \leq \mu_{6}\Leftrightarrow c_{1}\leq \theta_{6},\\ \mu \geq \mu_{7}\Leftrightarrow c_{1}\geq \theta_{12},\; & \mu \leq \mu_{9}\Leftrightarrow c_{1}\leq \theta_{13},\\ \mu \geq \mu_{10}\Leftrightarrow c_{1}\geq \theta_{14},\; & \mu \leq \mu_{13}\Leftrightarrow c_{1}\leq \theta_{15}. \end{array}$$

We divide the proof into several cases, first according to the values of $c_{1}$ and then according to the values of *n*.

Case 1: $\theta_{1}<c_{1}\leq 0$ . From (40), we have $\mu_{1}>\mu_{3}$ in this case and from (39), Φ(n,p,μ) simplifies to three cases:

$\Phi \left(n,p,\mu \right)\Leftrightarrow \max\left(\mu_{4},\theta_{7}\right)\leq \mu \leq \min\left(\mu_{2},\mu_{3}\right)$ , if $p\in (\frac{4}{9},n_{1})$ or $p>n_{2}$ ;

$\Phi \left(n,p,\mu \right)\Leftrightarrow \max\left(\mu_{2},\mu_{4},\theta_{7}\right)\leq \mu \leq \mu_{3}$ , if $p\in (n_{1},n_{2})$ ;

$\Phi \left(n,p,\mu \right)\Leftrightarrow \theta_{7}\leq \mu \leq \min\left(\mu_{2},\mu_{3}\right)$ or $\max\left(\mu_{4},\theta_{7}\right)\leq \mu \leq \min\left(\mu_{1},\mu_{2}\right)$ , if $p<\frac{4}{9}$ .

According to the vales of *p*, we consider seven cases below.

Case 1.1: $\theta_{1}<c_{1}\leq 0$ and $p>n_{2}$ . In this case, from (42) and (44), we have $\mu_{2}\geq \mu_{3}$ and $\mu_{4}>\theta_{7}$ . Hence, $\Phi \left(n,p,\mu \right)\Leftrightarrow \mu_{4}\leq \mu \leq \mu_{3}$ .

We now eliminate $c_{1}$ from $\Phi \left(n,p,\mu \right)\Leftrightarrow \left(p>n_{2}\wedge \theta_{1}<c_{1}\leq 0\wedge \mu_{4}\leq \mu \leq \mu_{3}\right)$ . By (49), $\mu_{4}\leq \mu \leq \mu_{3}$ is equivalent to $\theta_{5}\leq c_{1}\leq \theta_{4}$ . $\exists c_{1}(\theta_{5}\leq c_{1}\leq \theta_{4}\wedge \theta_{1}<c_{1}\leq 0$ ) is equivalent to ( $\theta_{4}>\theta_{1}\wedge \theta_{5}\leq 0$ ). Therefore, in this case, $\Phi \left(n,p,\mu \right)\Leftrightarrow \left(p>n_{2}\wedge \theta_{4}>\theta_{1}\wedge \theta_{5}\leq 0\right)$ , and T(1,2) is proved.

Case 1.2: $\theta_{1}<c_{1}\leq 0$ and $p=n_{2}$ . When $p=n_{2}$ , we have $\theta_{1}=\theta_{3},\;s_{2}=-\frac{1}{1024}\left(7\sqrt{17}-33\right)\left(c_{1}\sqrt{17}+64n+9c_{1}\right)n$ . Then, $s_{2}\geq 0\Leftrightarrow c_{1}\geq \theta_{3}$ . Because $\theta_{1}<c_{1}\leq 0$ and $p=n_{2}>\frac{4}{9}$ , we have $s_{3}\geq 0\Leftrightarrow \mu_{4}\leq \mu \leq \mu_{3}$ . By (44), we have $\mu_{4}>\theta_{7}$ . When $p=n_{2}$ , we have $\mu_{3}=\mu_{6}$ and $\mu_{4}=\mu_{7}$ . Thus $\Phi \left(n,p,\mu \right)\Leftrightarrow \left(\theta_{3}<c_{1}\leq 0,\mu_{7}\leq \mu \leq \mu_{6}\right)$ .

We now eliminate $c_{1}$ from $\Phi \left(n,p,\mu \right)\Leftrightarrow \left(p=n_{2}\wedge \theta_{3}<c_{1}\leq 0\wedge \mu_{7}\leq \mu \leq \mu_{6}\right)$ . By (49), $\mu_{7}\leq \mu \leq \mu_{6}$ is equivalent to $\theta_{12}\leq c_{1}\leq \theta_{6}$ . $\exists c_{1}\left(\theta_{12}\leq c_{1}\leq \theta_{6}\wedge \theta_{3}<c_{1}\leq 0\right)$ is equivalent to $\theta_{6}>\theta_{3}$ and $\theta_{12}\leq 0$ . Therefore, in this case, $\Phi \left(n,p,\mu \right)\Leftrightarrow \left(p=n_{2}\wedge \theta_{6}>\theta_{3}\wedge \theta_{12}\leq 0\right)$ , and T(2,2) is proved.

Case 1.3: $\theta_{1}<c_{1}\leq 0$ and $p\in (n_{1},n_{2})$ , p≠1 . This case is divided into two sub-cases.

Case 1.3.1: $\theta_{1}<c_{1}\leq 0$ and $p\in (\frac{13}{9},n_{2})$ . By (43) and (44), we have $\mu_{4}>\mu_{2}$ and $\mu_{4}>\theta_{7}$ . Hence, $\Phi \left(n,p,\mu \right)\Leftrightarrow \mu_{4}\leq \mu \leq \mu_{3}$ .

We now eliminate $c_{1}$ from $\Phi \left(n,p,\mu \right)\Leftrightarrow (p\in \left(\frac{13}{9},n_{2}\right)\wedge \theta_{1}<c_{1}\leq 0\wedge \mu_{4}\leq \mu \leq \mu_{3})$ . Similar to Case 1.1, we have $\Phi \left(n,p,\mu \right)\Leftrightarrow (p\in \left(\frac{13}{9},n_{2}\right)\wedge \theta_{4}>\theta_{1}\wedge \theta_{5}\leq 0)$ , T(3,2) is proved.

Case 1.3.2: $\theta_{1}<c_{1}\leq 0$ and $p\in (n_{1},\frac{13}{9}]$ , p≠1 . By (43)–(46), we have $\mu_{4}\geq \mu_{2}$ , ( $\mu_{3}\geq \theta_{7}\Leftrightarrow c_{1}\leq \eta_{1}$ ), ( $\mu_{4}\geq \theta_{7}\Leftrightarrow c_{1}\leq \theta_{2}$ ) and ( $\theta_{2}>\theta_{1}\Leftrightarrow \phi_{3}$ ). Hence $\Phi \left(n,p,\mu \right)\Leftrightarrow \max\left(\mu_{4},\theta_{7}\right)\leq \mu \leq \mu_{3}$ . This case is further divided into two sub-cases.

Case 1.3.2.1: If $c_{1}\leq \theta_{2}$ , then $\mu_{4}\geq \mu_{5}$ , and $\Phi \left(n,p,\mu \right)\Leftrightarrow \mu_{4}\leq \mu \leq \mu_{3}$ . Thus, we need $\theta_{1}<\theta_{2}$ , which yields $\phi_{3}$ . Thus, $\Phi \left(n,p,\mu \right)\Leftrightarrow \left(\theta_{1}<c_{1}\leq \theta_{2},\phi_{3},\mu_{4}\leq \mu \leq \mu_{3}\right)$ .

We now eliminate $c_{1}$ from $\Phi \left(n,p,\mu \right)\Leftrightarrow (p\in \left(n_{1},\frac{13}{9}\right),p\neq 1,\phi_{3},\theta_{1}<c_{1}\leq \theta_{2},\mu_{4}\leq \mu \leq \mu_{3})$ . Like Case 1.1, we have $\Phi \left(n,p,\mu \right)\Leftrightarrow (p\in \left(n_{1},\frac{13}{9}\right)\wedge p\neq 1\wedge \phi_{3}\wedge \theta_{4}>\theta_{1}\wedge \theta_{5}\leq \theta_{2})$ , and T(4,2) is proved

Case 1.3.2.2: If $c_{1}\geq \theta_{2}$ , then $\mu_{4}\leq \theta_{7}$ , and $\Phi \left(n,p,\mu \right)\Leftrightarrow \theta_{7}\leq \mu \leq \mu_{3}$ . Thus, we need $\theta_{7}\leq \mu_{3}$ , which yields $c_{1}\leq \eta_{1}$ . By (47), we know $\eta_{1}>\theta_{1}$ results in $\phi_{3}$ , which yields $\theta_{1}<\theta_{2}<\eta_{1}$ . Thus, $\Phi \left(n,p,\mu \right)\Leftrightarrow (\theta_{2}<c_{1}\leq \min\left(0,\eta_{1}\right),\phi_{3},\theta_{7}\leq \mu \leq \mu_{3})$ .

We now eliminate $c_{1}$ from $\Phi \left(n,p,\mu \right)\Leftrightarrow (p\in \left(n_{1},\frac{13}{9}\right),p\neq 1,\phi_{3},\theta_{2}<c_{1}\leq \min\left(0,\eta_{1}\right),\theta_{7}\leq \mu \leq \mu_{3})$ . $\theta_{7}\leq \mu \leq \mu_{3}$ is equivalent to $\theta_{7}\leq \mu$ and $c_{1}\leq \theta_{4}$ . $\exists c_{1}(c_{1}\leq \theta_{4}\wedge \theta_{2}<c_{1}\leq \min\left(0,\eta_{1}\right)$ ) is equivalent to $\theta_{4}>\theta_{2}$ . Therefore, in this case, $\Phi \left(n,p,\mu \right)\Leftrightarrow (p\in \left(n_{1},\frac{13}{9}\right)\wedge p\neq 1\wedge \phi_{3}\wedge \theta_{7}\leq \mu \wedge \theta_{4}>\theta_{2})$ , and T(4,3) is proved.

Case 1.4: $\theta_{1}<c_{1}\leq 0$ and $p=n_{1}$ . When $p=n_{1}$ , we have $\theta_{1}=\theta_{10},\;\theta_{2}=\theta_{11},\;\eta_{1}=\eta_{2},\;s_{2}=-\frac{1}{1024}\left(33+7\sqrt{17}\right)\left(c_{1}\sqrt{17}-64n-9c_{1}\right)n$ . Then $s_{2}\geq 0\Leftrightarrow c_{1}\geq \theta_{10}$ . Because $\theta_{1}<c_{1}\leq 0$ and $p=n_{1}>\frac{4}{9}$ , we have $s_{3}\geq 0\Leftrightarrow \mu_{4}\leq \mu \leq \mu_{3}$ . By (44), we have $\mu_{4}\geq \theta_{7}\Leftrightarrow c_{1}\leq \theta_{2}$ .

Case 1.4.1: Similar to Case 1.3.2.1, $\Phi \left(n,p,\mu \right)\Leftrightarrow \left(\theta_{1}<c_{1}\leq \theta_{2},\phi_{3},\mu_{4}\leq \mu \leq \mu_{3}\right)$ . When $p=n_{1}$ , we have $\mu_{3}=\mu_{9},\;\mu_{4}=\mu_{10},\;\phi_{3}\Leftrightarrow n<n_{3}$ . Thus $\Phi \left(n,p,\mu \right)\Leftrightarrow \left(\theta_{10}<c_{1}\leq \theta_{11},n<n_{3},\mu_{10}\leq \mu \leq \mu_{9}\right)$ .

We now eliminate $c_{1}$ from $\Phi \left(n,p,\mu \right)\Leftrightarrow \left(p=n_{1},\theta_{10}<c_{1}\leq \theta_{11},n<n_{3},\mu_{10}\leq \mu \leq \mu_{9}\right)$ . $\mu_{10}\leq \mu \leq \mu_{9}$ is equivalent to $\theta_{14}\leq c_{1}\leq \theta_{13}$ . $\exists c_{1}\left(\theta_{14}\leq c_{1}\leq \theta_{13}\wedge \theta_{10}<c_{1}\leq \theta_{11}\right)$ is equivalent to $\theta_{13}>\theta_{10}$ and $\theta_{14}\leq \theta_{11}$ . Therefore, in this case $\Phi \left(n,p,\mu \right)\Leftrightarrow \left(p=n_{1}\wedge n<n_{3}\wedge \theta_{13}>\theta_{10}\wedge \theta_{14}\leq \theta_{11}\right)$ , and T(5,2) is proved.

Case 1.4.2: Similar to Case 1.3.2.2, $\Phi \left(n,p,\mu \right)\Leftrightarrow (\theta_{2}<c_{1}\leq \min\left(0,\eta_{1}\right),\phi_{3},\mu_{5}\leq \mu \leq \mu_{3})$ . When $p=n_{1}$ , we have $\theta_{7}=\theta_{16}$ . Thus, $\Phi \left(n,p,\mu \right)\Leftrightarrow \left(\theta_{11}<c_{1}\leq \eta_{2},n<n_{3},\theta_{16}\leq \mu \leq \mu_{9}\right)$ .

We now eliminate $c_{1}$ from $\Phi \left(n,p,\mu \right)\Leftrightarrow \left(p=n_{1},\theta_{11}<c_{1}\leq \eta_{2},n<n_{3},\theta_{16}\leq \mu \leq \mu_{9}\right)$ . $\theta_{16}\leq \mu \leq \mu_{9}$ is equivalent to $c_{1}\leq \theta_{13}$ and $\mu \geq \theta_{16}$ . $\exists c_{1}\left(c_{1}\leq \theta_{13}\wedge \theta_{11}<c_{1}\leq \eta_{2}\right)$ is equivalent to $\theta_{13}>\theta_{11}$ . Therefore, in this case, $\Phi \left(n,p,\mu \right)\Leftrightarrow \left(p=n_{1}\wedge n<n_{3}\wedge \theta_{13}>\theta_{11}\wedge \mu \geq \theta_{16}\right)$ , and T(5,3) is proved.

Case 1.5: $\theta_{1}<c_{1}\leq 0$ and $p\in (\frac{4}{9},n_{1})$ . By (42) and (44), we have $\mu_{2}>\mu_{3}$ and ( $\mu_{4}\geq \theta_{7}\Leftrightarrow c_{1}\leq \theta_{2}$ ). Hence $\Phi \left(n,p,\mu \right)\Leftrightarrow \max\left(\mu_{4},\theta_{7}\right)\leq \mu \leq \mu_{3}$ .

Case 1.5.1: Similar to Case 1.3.2.1, we have $\Phi \left(n,p,\mu \right)\Leftrightarrow \left(\theta_{1}<c_{1}\leq \theta_{2},\phi_{3},\mu_{4}\leq \mu \leq \mu_{3}\right)$ .

We now eliminate $c_{1}$ from $\Phi \left(n,p,\mu \right)\Leftrightarrow (p\in \left(\frac{4}{9},n_{1}\right),\phi_{3},\theta_{1}<c_{1}\leq \theta_{2},\mu_{4}\leq \mu \leq \mu_{3})$ . Like Case 1.3.2.1, we have $\Phi \left(n,p,\mu \right)\Leftrightarrow (p\in \left(\frac{4}{9},n_{1}\right)\wedge \phi_{3}\wedge \theta_{4}>\theta_{1}\wedge \theta_{5}\leq \theta_{2})$ , and T(6,2) is proved

Case 1.5.2: Similar to Case 1.3.2.2, we have $\Phi \left(n,p,\mu \right)\Leftrightarrow \left(\theta_{2}<c_{1}\leq \eta_{1},\phi_{3},\mu_{5}\leq \mu \leq \mu_{3}\right)$ .

We now eliminate $c_{1}$ from $\Phi \left(n,p,\mu \right)\Leftrightarrow (p\in \left(\frac{4}{9},n_{1}\right),\phi_{3},\theta_{2}<c_{1}\leq \eta_{1},\theta_{7}\leq \mu \leq \mu_{3})$ . Like Case 1.3.2.2, we have $\Phi \left(n,p,\mu \right)\Leftrightarrow (p\in \left(\frac{4}{9},n_{1}\right)\wedge \phi_{3}\wedge \theta_{7}\leq \mu \wedge \theta_{4}>\theta_{2})$ , and T(6,3) is proved.

Case 1.6: $\theta_{1}<c_{1}\leq 0$ and $p=\frac{4}{9}$ . When $p=\frac{4}{9}$ , we have $\theta_{1}=\theta_{9},\;\eta_{1}=\eta_{3},\;\theta_{7}=\theta_{8},\;s_{3}=-\frac{4n}{6561}\left(162n+25c_{1}\right)\left(45n^{2}+81nu+25c_{1}\right)$ . Then $s_{3}\geq 0\Leftrightarrow \mu \leq \mu_{13}$ if $c_{1}\geq \theta_{9}$ . By (45), we know $\mu_{3}\geq \theta_{7}\Leftrightarrow c_{1}\leq \eta_{1}$ . And $\theta_{9}<\eta_{3}\Leftrightarrow n=1$ . Thus $\Phi \left(n,p,\mu \right)\Leftrightarrow \left(\theta_{9}<c_{1}\leq \eta_{3},n=1,\theta_{8}\leq \mu \leq \mu_{13}\right)$ .

We now eliminate $c_{1}$ from $\Phi \left(n,p,\mu \right)\Leftrightarrow \left(p=\frac{4}{9},\theta_{9}<c_{1}\leq \eta_{3},n=1,\theta_{8}\leq \mu \leq \mu_{13}\right)$ . $\theta_{8}\leq \mu \leq \mu_{13}$ is equivalent to $c_{1}\leq \theta_{15}$ and $\mu \geq \theta_{8}$ . $\exists c_{1}\left(c_{1}\leq \theta_{15}\wedge \theta_{9}<c_{1}\leq \eta_{3}\right)$ is equivalent to $\theta_{15}>\theta_{9}$ . Therefore, in this case $\Phi \left(n,p,\mu \right)\Leftrightarrow \left(p=\frac{4}{9}\wedge n=1\wedge \theta_{15}>\theta_{9}\wedge \mu \geq \theta_{8}\right)$ , and T(7,2) is proved

Case 1.7: $\theta_{1}<c_{1}\leq 0$ and $0<p<\frac{4}{9}$ .

Case 1.7.1: If we select $\theta_{7}\leq \mu \leq \min\left(\mu_{2},\mu_{3}\right)$ , by (42), we have $\mu_{2}>\mu_{3}$ . Thus, $\Phi \left(n,p,\mu \right)\Leftrightarrow \theta_{7}\leq \mu \leq \mu_{3}$ . So, we need $\theta_{7}\leq \mu_{3}$ , which yields $c_{1}\leq \eta_{1}$ . By (47), we know $\eta_{1}>\theta_{1}$ results in $\phi_{3}$ , which yields n=1 with $0<p<\frac{4}{9}$ . Thus $\Phi \left(n,p,\mu \right)\Leftrightarrow \left(\theta_{1}<c_{1}\leq \eta_{1},n=1,\theta_{7}\leq \mu \leq \mu_{3}\right)$ .

We now eliminate $c_{1}$ from $\Phi \left(n,p,\mu \right)\Leftrightarrow (p\in \left(0,\frac{4}{9}\right),n=1,\theta_{1}<c_{1}\leq \eta_{1},\theta_{7}\leq \mu \leq \mu_{3})$ . Like Case 1.3.2.2, we have $\Phi \left(n,p,\mu \right)\Leftrightarrow (p\in \left(0,\frac{4}{9}\right)\wedge n=1\wedge \theta_{7}\leq \mu \wedge \theta_{4}>\theta_{1})$ , and T(8,2) is proved.

Case 1.7.2: If we select $\max\left(\mu_{4},\theta_{7}\right)\leq \mu \leq \min\left(\mu_{1},\mu_{2}\right)$ , by (41), we have $\mu_{1}<\mu_{4}$ , which yields a contradiction.

Case 2: $c_{1}<\theta_{1}$ . From (40), we have $\mu_{1}<\mu_{3}$ in this case, and from (35), Φ(n,p,μ) simplifies to one case: $\Phi \left(n,p,\mu \right)\Leftrightarrow \theta_{7}\leq \mu \leq \min\left(\mu_{1},\mu_{2},\mu_{4}\right)$ , if $0<p<\frac{4}{9}$ and $c_{1}<\theta_{1}$ . Since *p* satisfies $0<p<\frac{4}{9}$ , we need only consider the following cases.

Case 2.1: $c_{1}<\theta_{1}$ and $0<p<\frac{4}{9}$ . By (41), (43) and (44), we have $\mu_{1}>\mu_{4}$ , $\mu_{2}>\mu_{4}$ and ( $\mu_{4}\geq \theta_{7}\Leftrightarrow c_{1}\geq \theta_{2}$ ). Then, we need $\theta_{2}<\theta_{1}$ , which yields $\phi_{3}$ by (46). Because $\phi_{3}$ means n=1 with $0<p<\frac{4}{9}$ , we have $\Phi \left(n,p,\mu \right)\Leftrightarrow \left(\theta_{2}\leq c_{1}<\theta_{1},n=1,\theta_{7}\leq \mu \leq \mu_{4}\right)$ .

We now eliminate $c_{1}$ from $\Phi \left(n,p,\mu \right)\Leftrightarrow (p\in \left(0,\frac{4}{9}\right),n=1,\theta_{2}\leq c_{1}<\theta_{1},\theta_{7}\leq \mu \leq \mu_{4})$ . $\theta_{7}\leq \mu \leq \mu_{4}$ is equivalent to $c_{1}\geq \theta_{5}$ and $\mu \geq \theta_{7}$ . $\exists c_{1}\left(c_{1}\geq \theta_{5}\wedge \theta_{2}\leq c_{1}<\theta_{1}\right)$ is equivalent to $\theta_{5}<\theta_{1}$ . Therefore, in this case, $\Phi \left(n,p,\mu \right)\Leftrightarrow (p\in \left(0,\frac{4}{9}\right)\wedge n=1\wedge \theta_{5}<\theta_{1}\wedge \mu \geq \theta_{7})$ , and T(8,3) is proved.

Case 2.2: $c_{1}<\theta_{1}$ and $p=n_{2}$ . In Case 1.5, we know that $\theta_{1}=\theta_{3}$ with $p=n_{2}$ , and $s_{2}\geq 0\Leftrightarrow c_{1}\geq \theta_{3}$ , which yields a contradiction.

Case 2.3: $c_{1}<\theta_{1}$ and $p=n_{1}$ . In Case 1.6, we know that $\theta_{1}=\theta_{10}$ with $p=n_{1}$ , and $s_{2}\geq 0\Leftrightarrow c_{1}\geq \theta_{10}$ , which yields a contradiction.

Case 2.4: $c_{1}<\theta_{1}$ and $p=\frac{4}{9}$ . We have $\theta_{1}=\theta_{9},\;\mu_{2}=\mu_{12},\;\mu_{3}=\mu_{13}$ based on $p=\frac{4}{9}$ . Then, we have ( $s_{2}\geq 0\Leftrightarrow \mu \leq \mu_{12}$ ) and ( $s_{3}\geq 0\Leftrightarrow \mu \geq \mu_{13}$ if $c_{1}\leq \theta_{9}$ ). So, we need $\mu_{12}\geq \mu_{13}$ . By (42), we have $\mu_{12}<\mu_{13}$ , which yields a contradiction.

Case 3: $c_{1}=\theta_{1}$ . When $c_{1}=\theta_{1}$ , we have $s_{1}=n\left(np-n-\mu +2\right)$ , $s_{2}=n\left(4p^{2}-9p+4\right)\left(np-n-\mu +2\right)$ and $s_{3}=-n^{2}\left(9p-4\right){\left(np-n-\mu +2\right)}^{2}$ . Thus, $s_{1}\geq 0\Leftrightarrow \mu \leq 2+n\left(p-1\right)$ and $s_{3}\geq 0\Leftrightarrow \left(p\leq \frac{4}{9}\;\mathrm{or}\;\mu =2+n\left(p-1\right)\right)$ .

Case 3.1: If μ=2+n(p−1) , then $s_{1}=s_{2}=s_{3}=0$ . Furthermore, $p\geq 1-\frac{\mu }{n}\Leftrightarrow \phi_{1}$ . Thus, $\Phi \left(n,p,\mu \right)\Leftrightarrow \left(c_{1}=\theta_{1},\phi_{1},\phi_{2}\right)$ , and T(i,4),i=1,…,6 are proved.

Case 3.2: If $p\leq \frac{4}{9}$ , then $s_{2}\geq 0\Leftrightarrow \mu \leq 2+n\left(p-1\right)$ . Then, we need $2+n\left(p-1\right)\geq \mu_{5}$ , which yields $\phi_{1}$ . And $\phi_{1}$ implies n=1 with $p\leq \frac{4}{9}$ . Thus, $\Phi \left(n,p,\mu \right)\Leftrightarrow \left(c_{1}=\theta_{1},n=1,-p\leq \mu -1\leq p\right)$ , and T(7,4) , T(8,4) are proved.

## 4. Conclusions

This paper is an extension of the work [15,16,17] to the case where the entropy power involves parameters. The basic idea is to prove entropy power inequalities in a systematic way. Precisely, the concavity of Rényi entropy power is considered, where the probability density $u_{t}$ solves the nonlinear heat equation with two parameters *p* and μ . Our procedure reduces the proof of the CREP to checking the semi-positiveness of a quadratic form (33) whose coefficients are polynomials in the parameters n,p,μ . In principle, a necessary and sufficient condition on parameters n,p,μ for this can be computed with the quantifier elimination [24]. Some interesting works [26,27] can help to understand our approach in this paper.

Based on the above method, we give a sufficient condition Φ(n,p,μ) for the CREP, which extends the parameter’s range of the CREP given by Savaré-Toscani [22]. By Theorem 3, our results give the necessary and sufficient condition for the CREP under certain conditions. However, in the general case, Theorem 1 only gives a sufficient condition for the following reasons: Problem 1 may not be equivalent to Problem 2, and more constraints may exist.

For future research, it is interesting to see whether the three conjectures about Costa’s differential entropy studied in [17] can be generalized to this more general case.

## Figures and Tables

**Table 1:** The description for Φ(n,p,μ) in (34).

$p>n_{2}$	$\theta_{4}>\theta_{1}\wedge \theta_{5}\leq 0$	*	$\phi_{1}\wedge \phi_{2}$
$p=n_{2}$	$\theta_{6}>\theta_{3}\wedge \theta_{12}\leq 0$	*	$\phi_{1}\wedge \phi_{2}$
$\frac{13}{9}<p<n_{2}$	$\theta_{4}>\theta_{1}\wedge \theta_{5}\leq 0$	*	$\phi_{1}\wedge \phi_{2}$
$n_{1}<p\leq \frac{13}{9}$ , p≠1	$\phi_{3}\wedge \theta_{4}>\theta_{1}\wedge \theta_{5}\leq \theta_{2}$	$\phi_{3}\wedge \theta_{7}\leq \mu \wedge \theta_{4}>\theta_{2}$	$\phi_{1}\wedge \phi_{2}$
$p=n_{1}$	$n<n_{3}\wedge \theta_{13}>\theta_{10}\wedge \theta_{14}\leq \theta_{11}$	$n<n_{3}\wedge \theta_{13}>\theta_{11}\wedge \mu \geq \theta_{16}$	$\phi_{1}\wedge \phi_{2}$
$\frac{4}{9}<p<n_{1}$	$\phi_{3}\wedge \theta_{4}>\theta_{1}\wedge \theta_{5}\leq \theta_{2}$	$\phi_{3}\wedge \theta_{7}\leq \mu \wedge \theta_{4}>\theta_{2}$	$\phi_{1}\wedge \phi_{2}$
$p=\frac{4}{9}$	$n=1\wedge \theta_{15}>\theta_{9}\wedge \mu \geq \theta_{8}$	*	n=1∧−p≤μ−1≤p
$0<p<\frac{4}{9}$	$n=1\wedge \theta_{7}\leq \mu \wedge \theta_{4}>\theta_{1}$	$n=1\wedge \theta_{5}<\theta_{1}\wedge \mu \geq \theta_{7}$	n=1∧−p≤μ−1≤p

## Data Availability

Not applicable.

## Appendix A. Constraints in (15)

In this appendix, we give the constraints in (15), where $u_{h_{1},h_{2}}=\frac{\partial^{h_{1}+h_{2}}u}{\partial^{h_{1}}x_{a,t}\partial^{h_{2}}x_{b,t}}$ .

$R_{1,a,b}=3pu_{1,0}^{4}+3uu_{2,0}u_{1,0}^{2}-5u_{1,0}^{4},$

$R_{2,a,b}=3pu_{0,1}^{4}+3uu_{0,2}u_{0,1}^{2}-5u_{0,1}^{4},$

$R_{3,a,b}=3pu^{2}u_{1,0}u_{3,0}+u^{3}u_{4,0}-3u^{2}u_{1,0}u_{3,0},$

$R_{4,a,b}=3pu^{2}u_{0,1}u_{3,0}+u^{3}u_{3,1}-3u^{2}u_{0,1}u_{3,0},$

$R_{5,a,b}=3pu^{2}u_{1,0}u_{2,1}+u^{3}u_{3,1}-3u^{2}u_{1,0}u_{2,1},$

$R_{6,a,b}=3pu^{2}u_{0,1}u_{2,1}+u^{3}u_{2,2}-3u^{2}u_{0,1}u_{2,1},$

$R_{7,a,b}=3pu^{2}u_{1,0}u_{1,2}+u^{3}u_{2,2}-3u^{2}u_{1,0}u_{1,2},$

$R_{8,a,b}=3pu^{2}u_{0,1}u_{1,2}+u^{3}u_{1,3}-3u^{2}u_{0,1}u_{1,2},$

$R_{9,a,b}=3pu^{2}u_{1,0}u_{0,3}+u^{3}u_{1,3}-3u^{2}u_{1,0}u_{0,3},$

$R_{10,a,b}=3pu^{2}u_{0,1}u_{0,3}+u^{3}u_{0,4}-3u^{2}u_{0,1}u_{0,3},$

$R_{11,a,b}=3pu_{0,1}u_{1,0}^{3}+3uu_{1,1}u_{1,0}^{2}-5u_{1,0}^{3}u_{0,1},$

$R_{12,a,b}=3pu_{0,1}^{3}u_{1,0}+3uu_{1,1}u_{0,1}^{2}-5u_{0,1}^{3}u_{1,0},$

$R_{13,a,b}=3pu_{0,1}^{2}u_{1,0}^{2}+2uu_{0,1}u_{1,0}u_{1,1}+uu_{1,0}^{2}u_{0,2}-5u_{1,0}^{2}u_{0,1}^{2},$

$R_{14,a,b}=3puu_{1,0}^{2}u_{2,0}+u^{2}u_{1,0}u_{3,0}+u^{2}u_{2,0}^{2}-4uu_{2,0}u_{1,0}^{2},$

$R_{15,a,b}=3puu_{0,1}u_{1,0}u_{2,0}+u^{2}u_{0,1}u_{3,0}+u^{2}u_{1,1}u_{2,0}-4uu_{2,0}u_{1,0}u_{0,1},$

$R_{16,a,b}=3puu_{1,0}^{2}u_{1,1}+u^{2}u_{1,0}u_{2,1}+u^{2}u_{1,1}u_{2,0}-4uu_{1,1}u_{1,0}^{2},$

$R_{17,a,b}=3puu_{0,1}u_{1,0}u_{2,0}+u^{2}u_{1,0}u_{2,1}+u^{2}u_{1,1}u_{2,0}-4uu_{2,0}u_{1,0}u_{0,1},$

$R_{18,a,b}=3puu_{0,1}^{2}u_{2,0}+u^{2}u_{0,1}u_{2,1}+u^{2}u_{0,2}u_{2,0}-4uu_{0,1}^{2}u_{2,0},$

$R_{19,a,b}=3puu_{0,1}u_{1,0}u_{1,1}+u^{2}u_{0,1}u_{2,1}+u^{2}u_{1,1}^{2}-4uu_{0,1}u_{1,0}u_{1,1},$

$R_{20,a,b}=3puu_{1,0}^{2}u_{0,2}+u^{2}u_{1,0}u_{1,2}+u^{2}u_{0,2}u_{2,0}-4uu_{1,0}^{2}u_{0,2},$

$R_{21,a,b}=3puu_{0,1}u_{1,0}u_{1,1}+u^{2}u_{1,0}u_{1,2}+u^{2}u_{1,1}^{2}-4uu_{0,1}u_{1,0}u_{1,1},$

$R_{22,a,b}=3puu_{0,1}^{2}u_{1,1}+u^{2}u_{0,1}u_{1,2}+u^{2}u_{0,2}u_{1,1}-4uu_{1,1}u_{0,1}^{2},$

$R_{23,a,b}=3puu_{0,1}u_{1,0}u_{0,2}+u^{2}u_{0,1}u_{1,2}+u^{2}u_{0,2}u_{1,1}-4uu_{0,2}u_{1,0}u_{0,1},$

$R_{24,a,b}=3puu_{0,1}u_{1,0}u_{0,2}+u^{2}u_{1,0}u_{0,3}+u^{2}u_{0,2}u_{1,1}-4uu_{0,2}u_{1,0}u_{0,1},$

$R_{25,a,b}=3puu_{0,1}^{2}u_{0,2}+u^{2}u_{0,1}u_{0,3}+u^{2}u_{0,2}^{2}-4uu_{0,2}u_{0,1}^{2},$

$R_{26,a,b}=3pu_{0,1}u_{1,0}^{3}+2uu_{2,0}u_{1,0}u_{0,1}+uu_{1,1}u_{1,0}^{2}-5u_{1,0}^{3}u_{0,1},$

$R_{27,a,b}=3pu_{0,1}^{2}u_{1,0}^{2}+uu_{0,1}^{2}u_{2,0}+2uu_{0,1}u_{1,0}u_{1,1}-5u_{1,0}^{2}u_{0,1}^{2},$

$R_{28,a,b}=3pu_{0,1}^{3}u_{1,0}+uu_{1,1}u_{0,1}^{2}+2uu_{0,2}u_{1,0}u_{0,1}-5u_{0,1}^{3}u_{1,0}.$
